# Prevalence, incidence, and factors associated with pain-related disabilities, and experiences of limitations due to pain among First Nations, Inuit, and Métis peoples in Canada: A scoping review

**DOI:** 10.17269/s41997-025-01047-z

**Published:** 2025-06-16

**Authors:** Astrid DeSouza, Dorothy Taylor, Jennifer L. Ward, Julie Vizza, Hainan Yu, Kent Murnaghan, Carol Cancelliere, Sheilah Hogg-Johnson, Amanda J. Sheppard, Pierre Côté

**Affiliations:** 1 Faculty of Health Sciences, Ontario Tech University, Oshawa, ON Canada; 2 Institute for Disability and Rehabilitation Research, Ontario Tech University, Oshawa, ON Canada; 3 Sacred Water Circle, Peterborough, ON Canada; 4 Curve Lake First Nation, Peterborough, ON Canada; 5 Natoaganeg Mi′kmaq Nation, Miramichi, NB Canada; 6 University of Manitoba, Winnipeg, MB Canada; 7 Department of Research and Innovation, Canadian Memorial Chiropractic College, Toronto, ON Canada; 8 Dalla Lana School of Public Health, University of Toronto, Toronto, ON Canada; 9 Indigenous Cancer Care Unit, Ontario Health, Toronto, ON Canada; 10 Library and Information Services, Canadian Memorial Chiropractic College, Toronto, ON Canada

**Keywords:** Pain, Disability, Indigenous health, Canada, Epidemiology, Douleur, Invalidité, Santé autochtone, Canada, Épidémiologie

## Abstract

**Objectives:**

To describe the prevalence, incidence, factors associated with pain-related disabilities, and experiences of limitations due to pain among First Nations, Inuit, and Métis peoples in Canada.

**Methods:**

We conducted a scoping review of the literature. The search strategy, developed with a health sciences librarian, included Indigenous-specific and health peer-reviewed databases, and grey literature for studies from inception to May 23, 2023. We included epidemiological, qualitative, and mixed-methods studies assessing pain-related disability outcomes among First Nations, Inuit, and Métis peoples in Canada.

**Synthesis:**

We screened 5902 citations from the peer-reviewed databases, of which 86 were screened as full-text items, and 49 were screened separately from grey literature sources. Two relevant items were retrieved. In 2017, an epidemiological study reported point prevalence estimates of pain-related disability lasting 6 months or more as follows: 11.4% among Inuit, 20.7% among Métis, and 22.2% among off-reserve First Nations people, with higher prevalence in women than in men. In 2002, a qualitative study highlighted emergent themes related to “difficulty coping with pain” and “suffering” among Cree adults with disabilities from the Mushkegowuk Territory. No studies reported on the incidence or factors associated with pain-related disability.

**Conclusion:**

Our scoping review found two studies on pain-related disabilities among Indigenous peoples in Canada. Continued collaboration with Indigenous partners is required to contextualize these findings and determine appropriate next steps.

**Supplementary Information:**

The online version contains supplementary material available at 10.17269/s41997-025-01047-z.

## Résumé

**Objectifs:**

Décrire la prévalence et l’incidence de l’invalidité attribuable à la douleur, les facteurs associés et les expériences de limitations dues à la douleur chez les membres des Premières Nations, les Inuits et les Métis au Canada.

**Méthode:**

Nous avons mené une étude de champ de la littérature. Notre stratégie de recherche, élaborée avec une bibliothécaire en sciences de la santé, incluait des bases de données examinées par les pairs portant sur les questions autochtones et sur la santé, ainsi que la littérature grise, pour les études menées jusqu’au 23 mai 2023. Nous avons inclus les études épidémiologiques, qualitatives et à méthodes mixtes qui évaluaient les résultats cliniques de l’invalidité attribuable à la douleur chez les membres des Premières Nations, les Inuits et les Métis du Canada.

**Synthèse:**

Nous avons sélectionné 5902 citations provenant des bases de données examinées par les pairs, dont 86 en texte intégral; nous en avons sélectionné 49 autres provenant de la littérature grise. Deux articles pertinents ont été récupérés. Une étude épidémiologique de 2017 présentait des estimations de la prévalence ponctuelle de l’invalidité attribuable à la douleur durant depuis six mois ou plus; cette prévalence était de 11,4% chez les Inuits, de 20,7% chez les Métis et de 22,2% chez les membres des Premières Nations hors des réserves; elle était plus élevée chez les femmes que chez les hommes. Une étude qualitative de 2002 dégageait des thèmes émergents liés à la «difficulté de gérer la douleur» et à la «souffrance» chez les adultes cris handicapés sur le territoire Mushkegowuk. Aucune étude ne portait sur l’incidence de l’invalidité attribuable à la douleur ni sur les facteurs associés.

**Conclusion:**

Notre étude de champ a relevé deux études sur l’invalidité attribuable à la douleur chez les peuples autochtones au Canada. Une collaboration soutenue avec des partenaires autochtones sera requise pour en contextualiser les constatations et déterminer les prochaines mesures qu’il convient de prendre.

## Introduction

Due to the devasting effects of colonization in Canada, First Nations, Inuit, and Métis peoples (henceforth referred to as Indigenous where applicable) are disproportionately affected by disability, with higher rates reported in Indigenous populations compared to non-Indigenous counterparts (Hahmann et al., 2019; Ng, 1996; Rivas Velarde, 2015). Living with disability can cause significant discomfort and pain, and conversely, pain itself can cause considerable disability. While disability and pain are closely interconnected, the body of literature regarding disability related to pain among First Nations, Inuit, and Métis peoples in Canada remains scarce. A recent scoping review identified the literature largely investigated pain conditions, while a review examining works in and outside of Canada reported a higher proportion of pain experienced by Indigenous peoples compared to non-Indigenous populations (Bailey et al., 2023; Jimenez et al., 2011). Although pain is highly prevalent (Barnabe et al., 2017; Hitchon et al., 2020; Latimer et al., 2018), little is known about the impact of pain on functioning, participation, and activities of daily living among First Nations, Inuit, and Métis peoples in Canada.

One factor contributing to this is that the construct of disability is dynamic and multifaceted. For many, it encompasses the relationship among body structures and functions, daily activities, and social participation, in interaction with personal and environmental factors (World Health Organization, 2001). As such, according to Western models, disability can arise when functioning is limited due to a health condition (World Health Organization, 2001). In contrast, among certain First Nations, Inuit, and Métis languages and communities, the term “disability” often does not exist or holds different meaning, and those born with different abilities are celebrated for their uniqueness (Indigenous Advisory Committee, personal communication, November 16, 2022; Roberts, 2022). For some, these abilities are meant to be viewed as gifts that serve a purpose in strengthening the community as a whole (Shackel, 2008; Stienstra et al., 2018). Hence, multiple realities can play a role in understanding the construct of disability. Moreover, the conceptualization of pain can extend beyond the context of physical sensations, interconnecting with emotional, social, and spiritual aspects of an individual (Barkwell, 2005). However, the predominance of Eurocentric knowledge paradigms has created a hierarchy between Western approaches and Indigenous knowledge. Thus, to understand pain-related disability among First Nations, Inuit, and Métis peoples in Canada, we must first consider the effects of historical and contemporary colonialism.

Indigenous peoples are those who are descendants of the original (first) inhabitants of the land (Government of Canada, 2024). Historically, the treatment of Indigenous peoples in Canada has been one of oppression, genocide, pain, exploitation, and racial discrimination increasing within the healthcare system (Allan & Smylie, 2015). Colonial processes have often resulted in a lack of Indigenous perspectives being part of decisions and policies pertaining to the health and well-being of First Nations, Inuit, and Métis, and urban Indigenous communities (Ladner, 2009). Living with disability often requires access to tailored community services, as well as health and rehabilitation services (Corso et al., 2022). However, Indigenous peoples with pain-related disabilities may face additional barriers to adequately accessing care. These barriers include, but are not limited to, discrimination and systemic racism, limited opportunities often resulting in financial barriers, limited access to and availability of health professionals, services and equipment, and scarcity of services in rural and urban areas (Rivas Velarde, 2015; Addressing Racism Review, 2020).

The effects of colonialism, the legacy of residential schools involving the forced removal of children from their communities, and the ban of cultural practices have had detrimental effects, which continue to be felt throughout multiple generations (Truth & Reconciliation Commission of Canada, 2015; Wilk et al., 2017; Woolford, 2009). Contemporary colonialism continues to prevail through the invasive control of non-Indigenous procedures, rules, and standards embedded within all levels of government. Alfred and Corntassel (2005) write of colonial processes that actively persist in “… trying to eradicate their existence as peoples through the erasure of the histories and geographies that provide the foundation for Indigenous cultural identities and sense of self” (p. 598). As such, colonialism has led to an unequal distribution of resources, supports, and power that has given rise to differences in opportunities available for First Nations, Inuit, and Métis peoples. This has created barriers towards achieving optimal health, particularly for those with pain-related disabilities. In their 2020 report, the Canadian Pain Task Force describes a higher incidence of pain and pain-related disability among Indigenous peoples than in non-Indigenous populations. The report further describes symptoms related to chronic pain as a primary reason for seeking healthcare among Indigenous peoples; however, due to systemic racism and discrimination within the healthcare system, many Indigenous people do not get the care they need (Canadian Pain Task Force, 2020). As such, for those living with pain-related disabilities, these barriers to seeking and receiving healthcare can have profound implications on the health and well-being of Indigenous peoples who may be faced with an unsafe environment and fearful to seek necessary care when needed (Graham et al., 2023; Monchalin et al., 2020).

For too long, Indigenous voices and perspectives have been excluded from conversations pertaining to their own health (Corso et al., 2022). Investigating pain-related disabilities through respectful and active engagement with First Nations, Inuit, and Métis peoples further supports the underpinnings on which the 94 calls to action issued by the Truth and Reconciliation Commission of Canada (2015) were built. Exploring this area of research offers insights into a novel field of literature, allowing us to significantly contribute towards understanding the epidemiology of pain-related disabilities through an evidence-informed dialogue with Indigenous peoples. This in turn can contribute towards developing effective and equitable healthcare access, delivery, and services grounded in community priorities to reduce pain-related disabilities. Working collaboratively with an Indigenous Advisory Committee, and guided by the Mi'kmaq principles of *Etuaptmumk* (Two-Eyed Seeing) in which both Indigenous and Western knowledge and ways of knowing are used (Marshall et al., 2018), we conducted a scoping review to identify and synthesize the peer-reviewed and grey literature to describe the prevalence, incidence, and factors associated with pain-related disabilities, and experiences of limitations in activities, functioning, or participation due to pain among First Nations, Inuit, and Métis peoples in Canada.

## Review questions

Our scoping review addressed the following research questions: (1) What is the prevalence of disability related to pain among Indigenous peoples in Canada?; (2) What is the incidence of disability related to pain among Indigenous peoples in Canada?; (3) What factors are associated with pain-related disabilities among Indigenous peoples in Canada?; and (4) What are the experiences of limitations in activities, functioning, or participation due to pain among Indigenous peoples in Canada?

## Inclusion criteria

### Population

We included studies of peoples from First Nations, Inuit, Métis, and urban Indigenous nations and communities of any age in Canada. If the study included Indigenous and non-Indigenous participants, stratified results by Indigenous identity needed to be available.

### Concept

Eligible studies investigated pain-related disabilities, which were defined as experiencing limitations in functioning, daily activities, or participation due to pain (World Health Organization, 2001). This included disability related to any conditions associated with pain, self-reported or medically diagnosed. While pain can present in any form (e.g. physical, emotional, mental, or spiritual), it had to be linked to disability to be eligible for our review. We excluded studies which focused on the prevalence or incidence of pain-related conditions (e.g. rheumatoid arthritis) that did not report on pain-related disability.

### Context

Studies conducted in Canada during any year or within any setting which reported on the prevalence, incidence, factors, or experiences associated with pain-related disabilities were included.

### Types of sources

With the specificity of the research questions above, eligible studies met the following criteria: (1) epidemiological studies (cross-sectional studies to estimate prevalence or associated factors; cohort or ecologic studies to estimate prevalence, incidence or associated factors; case–control studies to estimate associated factors); (2) qualitative studies to describe experiences of limitations in activities, functioning, or participation due to pain; we also included qualitative studies to inform the analysis of epidemiological studies regarding variables to consider when understanding potential factors associated with pain-related disabilities; or (3) mixed-methods studies. We excluded the following publication types from the overall evidence synthesis: case series, case reports, narrative reviews and systematic reviews, scoping reviews, randomized controlled trials, commentaries, guidelines, letters, editorials, books and book chapters, meeting abstracts, lectures, cadaveric or animal studies. However, we manually reviewed the reference lists of relevant narrative, scoping and systematic reviews, conference abstracts, and book chapters to determine whether any articles included may be eligible in answering our research questions.

## Methods

We selected a scoping review as a starting point to understand the breadth and depth of the current landscape of the pain-related disability literature among Indigenous peoples in Canada, and identify the type of evidence available and gaps in knowledge to inform future research initiatives (Munn et al., 2018). The conduct of our scoping review was guided by the JBI methodology for scoping reviews (Peters et al., 2020) and according to the Preferred Reporting Items for Systematic Reviews and Meta-analyses Extension for Scoping Reviews (PRISMA-ScR) Checklist (Tricco et al., 2018). Our scoping review protocol was registered a priori with Open Science Framework (OSF) Registries (10.17605/OSF.IO/TSH5W).

### Search strategy

We developed our search strategy with an experienced health sciences librarian, along with a second librarian who independently reviewed our strategy using the Peer Review of Electronic Search Strategies Checklist (McGowan et al., 2016). We searched both Indigenous-specific (Informit Indigenous Collection) and health peer-reviewed databases—MEDLINE (Ovid), EMBASE (Ovid), CINAHL (EBSCO), PubMed, PsycINFO (Ovid), and Scopus for epidemiological, qualitative, and mixed-methods studies—with no limits placed on publication years or language. We defined keyword search terms and concept groups related to pain, disability, and Indigenous peoples in Canada. The initial search strategy was developed using subject headings specific to Ovid MEDLINE and then adapted to the other databases (Appendix A).

We followed the methodology proposed in the *Cochrane Handbook* to identify relevant grey literature, and as such searched reports, dissertations, and theses (through key organizations, websites, and ProQuest Dissertations and Theses Global database) (Lefebvre et al., 2022). When searching the grey literature, we used Grey Matters Checklist (CADTH, 2019), along with other documentation strategies to guide the conduct of our grey literature search for published and unpublished studies (Fuller & Lenton, 2018). Appendix B outlines the detailed strategies used to organize the grey literature search.

### Study/source of evidence selection

We used a two-phase screening process (phase I: titles/abstracts and phase II: full-text) to identify eligible studies and literature. Prior to phase I, AD, JV, and HY pilot tested the screening of titles/abstracts with 100 randomly selected citations to discuss any disagreements or inconsistencies prior to screening. Reviewers needed to achieve ≥ 80% agreement during pilot testing before completing title/abstract screening. Pairs of trained reviewers independently screened all items (AD/JV, and AD/HY) in phase I and phase II to identify relevant studies. We similarly screened the grey literature in a two-phase process. Any disagreements throughout the peer-reviewed database and grey literature screening process were discussed, with the paired reviewers reaching consensus. We used EPPI-Reviewer (version 6.15.0.2) to manage all phases of the review process (Thomas et al., 2022). Full-text studies which did not meet the inclusion criteria were excluded, with reasons for their exclusion provided in Appendix C.

### Data extraction

One reviewer extracted data from eligible studies and built evidence tables. We extracted the following information from relevant studies: author, year, years of observation, study aim, design and mode of administration, sample participants, age, sex/gender, sociodemographic and health characteristics, Indigenous population or community, geographical region, pain-related disability outcome measure, comparison population, and adjustment variables. We extracted estimates of prevalence and incidence, and measures of association for associated factors (where applicable and provided). We also extracted themes in relation to describing limitations in activities, functioning, or participation due to pain. The data extracted were verified independently by a second reviewer.

### Data analysis and presentation

We computed the inter-rater reliability using the Cohen’s kappa statistic and percentage of agreement between reviewers for the titles/abstract and full-text screening. From the charted data, we narratively summarized the characteristics of the included studies organized by data first describing epidemiological studies on the prevalence of pain-related disability, the incidence of developing a pain-related disability, and factors associated. Next, we provided relevant information from qualitative (e.g., themes) and mixed-methods studies on individuals reporting limitations in activities, functioning, or participation due to pain.

### Indigenous Advisory Committee

We conceptualized our scoping review using elements of an integrated knowledge translation approach (Canadian Institutes of Health Research (CIHR), 2015). An Indigenous Advisory Committee (IAC) was established and consisted of a respected Elder and traditional knowledge holder, and Indigenous healthcare professionals and researchers, who are knowledge-users themselves, with expertise within the realm of Indigenous health and well-being. The IAC worked collaboratively with the research team in the development of the review methodology to review the research questions, concept groups, and key search terms, as well as the search strategy. The IAC had representation from First Nations, Métis, rural, and urban Indigenous individuals, all of whom resided in different geographical areas within Canada. In addition, the IAC provided guidance on additional databases and sources of grey literature to be searched, reviewed materials, and provided reflections on the included studies, and guidance on knowledge translation and next steps. The teachings and reflections shared influenced our thinking and shaped the interpretation of our findings. With each Indigenous community having its own identity, history, and culture, we acknowledge that while it was not possible to engage members from all communities, we strive to work with and identify a diverse group of individuals who brought complementary knowledge to the IAC in consideration of the physical, mental, emotional, and spiritual dimensions of health and functioning.

In collaboration with the IAC, the findings of this review were discussed with its members in order to contribute towards the development of an evidence-informed program of research to further understand pain and disability among Indigenous peoples in Canada. During meetings with the IAC, the study protocol, plan for analysis, and interpretation of results were discussed. In addition to working with the IAC to discuss the dissemination of findings through appropriate knowledge translation activities, we consulted with the Canadian Chiropractic Association (CCA), a national organization which works to address and advocate for the musculoskeletal health needs of all, to actively share our findings with Canadian chiropractors and chiropractic organizations. We also worked with the IAC and CCA to identify the implications of the findings for healthcare professionals, along with next steps.

### Study protocol deviation

Our review protocol registered on OSF Registries indicated we would explore disability related to painful conditions, specifically: (1) What is the prevalence of disability related to painful conditions among Indigenous peoples in Canada?; (2) What is the incidence of disability related to painful conditions among Indigenous peoples in Canada?; and (3) What factors are associated with pain-related disabilities among Indigenous peoples in Canada? However, after consultations with the IAC, our research questions 1 and 2 were modified prior to the review being initiated to state “disability related to pain” in lieu of “painful conditions”. This revision was made because experiencing pain-related disability may not solely be restricted to those with a painful condition. Additionally, given that our search included qualitative studies, a fourth objective was added to clarify the purpose of including qualitative study designs within the research objectives stated.

We updated our scoping review methodology to follow the guidance provided by the most recent *JBI Manual for Evidence Synthesis* (Peters et al., 2020), which builds on the six-stage approach described by Arksey and O’Malley and recommendations by others (Arksey & O’Malley, 2005; Levac et al., 2010; O’Brien et al., 2016). As such, a preliminary search for existing and in-progress scoping or systematic reviews on the topic was not identified through the Cochrane Database of Systematic Reviews and JBI Evidence Synthesis. Moreover, because our initial screening yielded only one epidemiological study, we re-screened the 829 citations that were initially excluded based on study design to include case reports and case-series (hypothesis-generating designs), and theses. As a result, we identified one (Master’s thesis) that met our inclusion criteria.

## Results

**Fig. 1:**
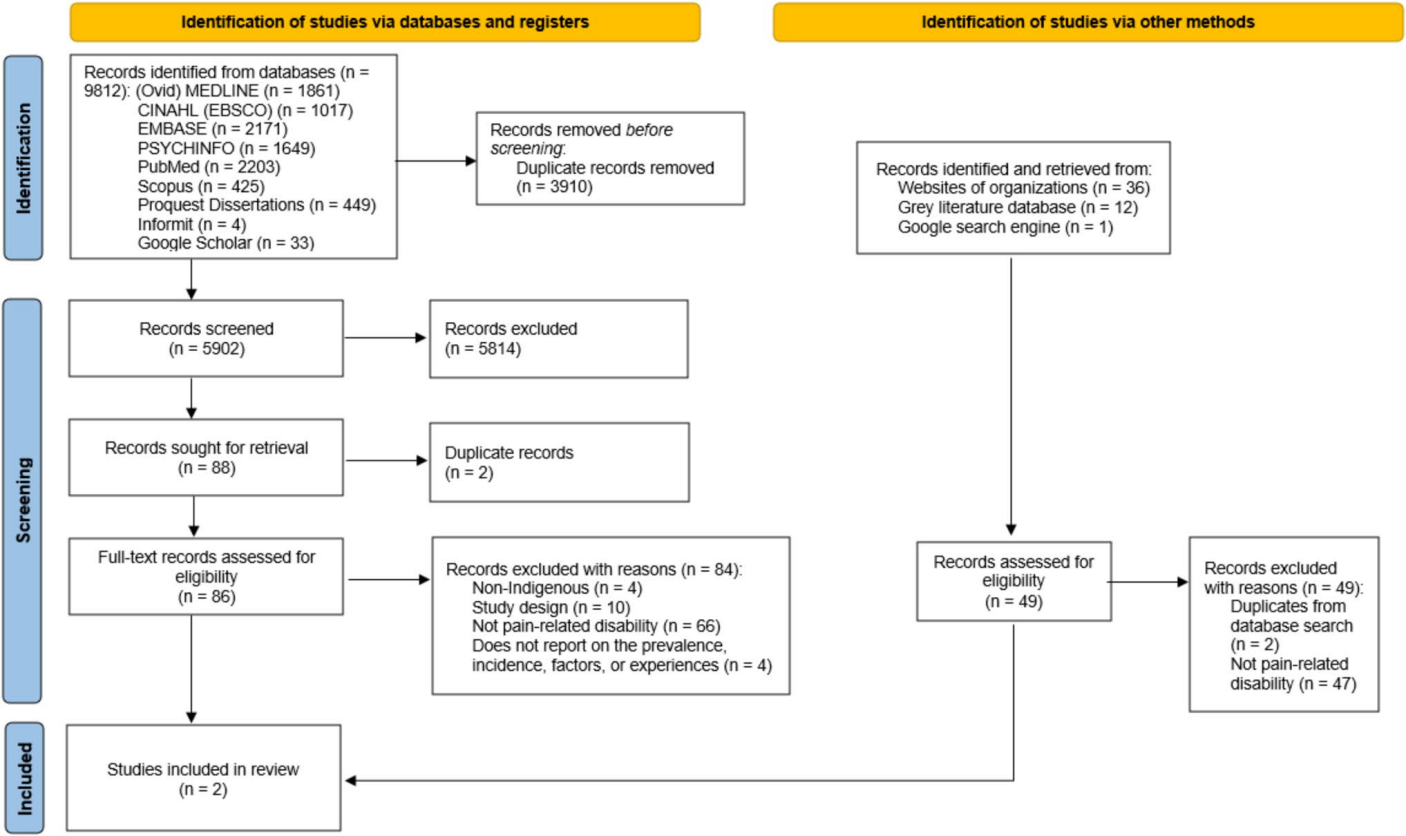
Preferred Reporting Items for Systematic Reviews and Meta-analyses (PRISMA) flow diagram for scoping reviews showing identification and selection of included studies

We retrieved 9812 citations from the peer-reviewed literature of which 86 full-text items were reviewed for eligibility, and 49 from the grey literature (Fig. 1). For the titles/abstracts screened by paired reviewers, the percentage of agreement was greater than 95% with corresponding kappas of 0.55 [95% CI (0.42, 0.68)] and 0.76 [95% CI (0.66, 0.86)] for the two reviewing pairs. Screening of full-text items in phase II yielded perfect agreement. Of those, two were included in our review: a national cross-sectional study which reported the prevalence of pain-related disabilities among First Nations people living off reserve, Métis and Inuit (*objective 1*) (Hahmann et al., 2019), and a qualitative study which explored the lived experiences of Cree adults living with disabilities from the Mushkegowuk Territory (*objective 4*) (Robarts, 2002). No studies reported the incidence of pain-related disability, or factors associated (*objectives 2 and 3*).

During full-text screening, we excluded most citations because they did not report on pain-related disability (Appendix C).

## Epidemiological studies

### The prevalence of pain-related disabilities

The cross-sectional study conducted by Statistics Canada (Hahmann et al., 2019) used the Disability Screening Questions (DSQ) introduced in the 2017 Aboriginal Peoples Survey to measure the prevalence of disability among First Nations people living off reserve, Métis and Inuit 15 years and older. The authors reported that the point prevalences of pain-related disabilities lasting 6 months or more were 11.4% among Inuit, 20.7% among Métis, and 22.2% among First Nations people living off reserve (Hahmann et al., 2019) (Table 1). Within this study, the DSQ asked about any form of pain. The prevalence estimates for First Nations people living off reserve and Métis were higher than those for the non-Indigenous comparison group (14.2%).

**Table 1:** Data extraction table for scoping review on the prevalence, incidence, and factors associated with pain-related disabilities, and experience of limitations due to pain among First Nations, Inuit, and Métis peoples in Canada

Publication	Study characteristics				Sample characteristics			Outcome measure	Comparison population	Pain-related disability estimates
	Years of observation	Aim	Design and mode of administration	Recruitment and sample	Age	Sex, gender	Indigenous group			
Hahmann et al., 2019	Data collection period: January 16 – August 15, 2017	To provide an overview of disability prevalence estimates among First Nations people living off reserve, Métis, and Inuit	Cross-sectional Computer-assisted personal interviews and computer-assisted telephone interviews	Complex multiple-phase stratified random sampling design *Indigenous participants who completed the survey: *n* = 23,946	15 years and older	Not provided	First Nations people living off reserve, Métis, and Inuit Throughout Canada	Disability Screening Questions (DSQ) within the 2017 Aboriginal Peoples Survey	Non-Indigenous	Prevalence: First Nations people living off reserve *22.2%* *[95% CI not provided]* Métis *20.7%* *[95% CI not provided]* Inuit *11.4%* *[95% CI not provided]* Sex-specific: First Nations people living off reserve Men *17.4% [95% CI not provided]* Women *26.2% [95% CI not provided]* Métis Men *17.2% [95% CI not provided]* Women *24.0% [95% CI not provided]* Inuit Men *8.8% [95% CI not provided]* Women *13.7% [95% CI not provided]*
Robarts, 2002	Data collection period: September to December 2001	To explore the lived experiences and understand the needs of Cree adults with disabilities living in remote communities of the Mushkegowuk Territory	Ethnography Focus groups, semi-structured interviews and participant observation Facilitated visual techniques with participants, such as impact drawings, social mapping, fish and rocks drawing exercise, flowcharts, and Venn diagrams	Volunteer, nominative, and snowballing strategies Persons with disabilities (*n* = 31) Healthcare professionals and community spokespersons (*n* = 15) Caregivers (*n* = 15)	28–88 years Not available 20–60 years	Male (n = 13) Female (n = 18) Not available Not available	Cree Mushkegowuk Territory (4 remote communities: Moosonee, Moose Factory, Attawapiskat, and Kashechewan)	**Themes	Not applicable	Themes related to pain-related disability for participants living with disabilities: Difficulty coping with pain Suffering

^*^Information obtained from supplementary documentation. Eligible Indigenous respondents: 34,176, and Indigenous participants who completed the survey: *n* = 23,946. Response rate: 70.1% (response rate during collection includes the total number of respondents divided by the total number of eligible units)

^**^This thesis presents the following emergent themes from case descriptions of select Mushkegowuk Territory community members living with disabilities: (1) ineffective help-seeking behaviour (waiting for help, not asking for help); (2) ineffective/inadequate community response to disability; (3) difficulty coping with pain; (4) isolation; (5) suffering; and (6) losing hope

In addition, the single cross-sectional study reported that women reported a higher prevalence of pain-related disabilities as compared with men (Hahmann et al., 2019). This finding was consistent among the three groups, First Nations people living off reserve (women 26.2%, men 17.4%), Métis (women 24.0%, men 17.2%), and Inuit (women 13.7%, men 8.8%). The prevalence estimates for First Nations people living off reserve and Métis were higher than those for the non-Indigenous comparison group (women 16.1%, men 12.2%).

### The incidence of pain-related disabilities

No studies reported the incidence of pain-related disability.

### Factors associated with pain-related disabilities

No studies reported factors associated with pain-related disability.

## Qualitative and mixed-method studies

### The experiences of limitations in activities, functioning, or participation due to pain

Robarts (2002) conducted a qualitative ethnographic study using participatory development methods to understand the lived experiences of Cree adults with disabilities from communities in the Mushkegowuk Territory. Focus groups and semi-structured interviews were conducted with 31 people with disabilities, 15 caregivers, and 15 community spokespersons and healthcare professionals. From the case descriptions of individuals living with disabilities, two (of the six) emergent themes spoke towards experiences of limitations in activities, functioning, or participation due to pain. These two themes were “Difficulty coping with pain” and “Suffering”. Additionally, the impact of pain was an underlying notion throughout this thesis.

## Discussion

To our knowledge, this article is the first comprehensive review in Canada to investigate the peer-reviewed and grey literature exploring the prevalence, incidence, and factors associated with pain-related disabilities, and any experiences of limitations in activities, functioning, or participation due to pain among First Nations, Inuit, and Métis peoples. Our scoping review found only two studies: one with pain-related disability prevalence estimates for First Nations out of community, Métis, and Inuit from different geographical locations throughout Canada, and another which shed light on important themes with regard to the impact of pain on functioning for Cree adults living with disabilities from communities in the Mushkegowuk Territory. No studies reporting on the incidence or factors associated were found.

Previous systematic reviews summarized the measures of occurrence for pain-related conditions such as rheumatic diseases and multiple sclerosis among Indigenous populations in the Americas (Canada, the United States, Latin America), Australia, and New Zealand (McDougall et al., 2017; Robers et al., 2023). Further reviews explored pain without focusing on disability or functioning. For example, Jimenez et al. (2011) found 12 studies which focused on the epidemiology of pain among American Indian, Alaska Native, and Aboriginal peoples, two of which reported prevalence estimates for painful conditions, such as arthritis among adult First Nations in the province of Manitoba, and dental caries among First Nation preschool children in the Inuvik Region. Additionally, Julien et al.’s (2018) review of the non-cancer pain literature identified ten studies that reported prevalence estimates of chronic non-cancer pain in general or condition-specific among Aboriginal people in Canada. However, a key feature differentiating our review from those previous ones is that these reviews did not focus on the disability experienced due to pain (i.e. limitations in functioning or activities, or participation restrictions).

In addition, through our discussions with the IAC, the few studies retrieved further reinforced Indigenous perspectives that their pain continues to be minimized, not taken seriously, nor validated in the research literature. It remains largely unknown whether current outcome measures used for pain are reflective of the true pain experienced by Indigenous peoples given that pain manifests itself in many different forms, from physical to emotional, mental, and spiritual. Given the low number of studies in our review, we hypothesize the subjective nature of pain and the diverse understanding of disability within First Nations, Inuit, and Métis communities as a contributing factor to the current gap in the literature. Therefore, the findings of our scoping review highlight the importance for healthcare professionals to acknowledge and recognize the different ways pain and disability can be expressed. Accounting for these differences can prevent experiences of pain and disability from being devalued, dismissed as inconsequential, minimized, or misinterpreted.

### Strengths and limitations

Our scoping review, developed in collaboration with the Indigenous Advisory Committee, takes a strength-based and decolonized approach to centre Indigenous voices. Other strengths include using a systematic methodology to search the literature and working with a health sciences librarian to develop a robust search strategy, which was peer-reviewed by a second librarian. We conducted a comprehensive search of various databases, including both peer-reviewed and grey literature. Furthermore, we utilized the health science search filters by the University of Alberta library, which contain an extensive set of Indigenous-specific search filters per province and territory in Canada (University of Alberta, 2023). All reviewers underwent training with a pilot screening exercise prior to commencing the phase I title and abstract screening.

We also acknowledge the limitations of our scoping review. Our selection criteria focused on studies conducted in Canada and on studies that investigated pain-related disability. Therefore, the results of our review may not be generalizable to other Indigenous populations. The concepts of “pain” and “disability” have varying meanings among different Indigenous communities, which may result in the potential omission of community-specific terminology used to describe these concepts. Furthermore, given the diversity among First Nations in and out of community, Métis, and Inuit groups, and urban Indigenous peoples, it is possible that certain terms used to describe specific nations, communities, bands, or tribes may not have been captured. To mitigate this, we worked with members of the IAC during the development of the search strategy to integrate Indigenous terminology and perspectives from the onset. Finally, while the IAC had representation from First Nations, Métis, and urban Indigenous individuals, this committee did not include direct representation from Inuit communities, despite our efforts.

## Implications for research, practice, and policy

Although the prevalence of pain-related disability among Indigenous peoples in Canada is not well documented, it is known that the prevalence of other types of disabilities and overall disability is higher among Indigenous peoples than among non-Indigenous populations (Hahmann et al., 2019; Ng, 1996). Moreover, the study by Hahmann et al. (2019) included in our scoping review found that pain-related disabilities were the most prevalent type of disability among the ten different types measured in the 2017 Aboriginal Peoples Survey.

Given these findings, we make a specific call to action for those involved in Indigenous health research and care. It is crucial to recognize the importance of incorporating First Nations, Inuit, and Métis methodologies and perspectives (King et al. 2009). Researchers, healthcare providers, and policymakers must work collaboratively *with* and *for* communities to address and remove barriers to care and improve access to supports and services. For researchers, this means designing studies that are culturally relevant and inclusive of Indigenous ways of knowing. For healthcare providers, it involves being aware of and sensitive to the unique experiences and needs of Indigenous patients with pain-related disabilities. For policymakers, it is essential to develop policies that support equitable access to healthcare and resources for Indigenous communities. By working together and prioritizing the inclusion of Indigenous voices and knowledge systems, we can develop more effective and equitable healthcare practices and policies that reduce pain-related disabilities and improve overall health outcomes for First Nations, Inuit, and Métis peoples.

## Conclusion

Our scoping review identified two studies addressing pain-related disabilities among First Nations, Inuit, and Métis peoples in Canada. The limited number of studies highlights a substantial knowledge gap. Addressing this research gap is urgent. Researchers, healthcare professionals, and policy makers must move beyond colonial frameworks and actively engage with Indigenous communities to co-create knowledge and develop culturally relevant solutions. In doing so, we can gain a more accurate understanding of the true extent of pain-related disabilities and work towards more effective and equitable healthcare practices and policies, ultimately improving health outcomes for Indigenous populations across Canada.

## Contributions to knowledge

What does this study add to existing knowledge?

- Our scoping review highlights a substantial knowledge gap in understanding the epidemiology of pain-related disabilities among Indigenous peoples in Canada.
- From the two studies retrieved, though the quantitative study helped provide prevalence estimates to understand how many may be living with pain-related disabilities, the qualitative study sheds light on other study designs which could potentially inform this area of research.

What are the key implications for public health interventions, practice, or policy?

- Given the lack of evidence available in the literature, we must collaboratively work with Indigenous communities, grassroots organizations, and those with lived experiences to co-design public health intervention, practices, and policies which support those living with pain-related disabilities, enable our knowledge to grow, and continue to move forward this important dialogue.

## Supplementary Information

Below is the link to the electronic supplementary material which contains appendices.

Supplementary file1 (DOCX 86 KB)

## Data Availability

Detailed search strategy provided in the supplementary material Appendix A.

## Code availability

EPPI-Reviewer version 6.15.0.2 to manage all studies throughout the review process.
