# Supplementary material for: Prevalence, incidence, and factors associated with pain-related disabilities, and experiences of limitations due to pain among First Nations, Inuit, and Métis peoples in Canada: A scoping review
Source: Can J Public Health. 2025 Jun 16;117(1):74–85. doi: 10.17269/s41997-025-01047-z (PMC12992870; doi:10.17269/s41997-025-01047-z)
Supplement: Supplementary file 1 — Supplementary file1 (DOCX 86 KB) [file 41997_2025_1047_MOESM1_ESM.docx]

| CMCC Library | Prevalence, incidence and factors associated with pain-related disability among Indigenous peoples in Canada: a scoping review |
| --- | --- |

**Appendix A: Search strategy**

**Database & Platform:**  Ovid MEDLINE

**Years of search:** Inception - current           **Languages**:      No limit

**Date search run:** APRIL 5 2023

**Number of records retrieved:** 1845

Database(s): Ovid MEDLINE(R) and Epub Ahead of Print, In-Process, In-Data-Review & Other Non-Indexed Citations, Daily and Versions

1 Indigenous Peoples/

2 Health Services, Indigenous/

3 Indians, North American/

4 Inuits/

5 Indigenous Canadians/

6 (indigenous* or aboriginal* or "first nations" or "first nation" or inuit* or inuk* or metis* or michif* or "Native people" or "native peoples").mp.

7 exp Canada/

8 ((turtle* adj island*) or canad* or nunavut* or yukon* or (northwest adj1 territor*) or (british adj1 columbia*) or alberta* or saskatchewan* or manitoba* or ontario* or quebec* or new brunswick or prince edward island or nova scotia or newfoundland or toronto* or vancouver* or montreal* or ottawa* or calgary* or edmonton* or winnipeg* or regina* or hamilton* or halifax*).mp.

9 1 or 2 or 3 or 4 or 6

10 7 or 8

11 9 and 10

12 5 or 11

13 (((exp Indians, North American/ or exp Inuits/ or exp Health Services, Indigenous/ or exp Ethnopharmacology/ or (exp Medicine, Traditional/ not Chinese.mp.) or exp Shamanism/ or (Athapaskan or Saulteaux or Wakashan or Cree or Aboriginal* or Indigenous* or Metis or "off-reserve" or "on reserve" or First Nation or First Nations or Amerindian or (urban adj3 (Indian* or Native* or Aboriginal*)) or ethnomedicine or country food* or residential school* or shaman* or traditional medicine* or traditional heal* or traditional food* or medicine man or medicine woman or autochtone* or (Native adj1 (man or men or women or woman or boy* or girl* or adolescent* or youth or youths or person* or adult or people* or Indian* or Nation or tribe* or tribal or band or bands))).mp.) and (exp British Columbia/ or (British Columbia or Colombie Britannique or Williams Lake or Vernon or White Rock or Salmon Arm or Quesnel or Powell River or Port Moody or Port Hardy or Port Coquitlam or Port Albernie or Pitt Meadows or Penticton or New Westminister or Nanaimo or Kelowna or Kamloops or Fort St John or Fernie or Enderby or Dawson Creek or Coquitlam or Chilliwack or Campbell River or Tumbler Ridge or Skidegate or Sandspit or Queen Charlotte or Port Clements or Kitimat or Hudsons Hope or Haida Gwaii or Fort St James or Fort Nelson or Dease Lake or Pouce Coupe or Chetwynd or Gransile or Atlin or Alexis Creek or Sooke or Cache Creek or Chehalis or Cheslatta or Kingcome Inlet or Kitwanga or Iskut or Kyuqot or Kanaka or Clo-oose or Nicomen or Liard River or Mount Currie or Keremos or Matsqui or Nanaimo or Tahsis or Gitwinkshilkw or Osoyoos or Popkum or Sechelt or Skookumchuk or (Siska not Karol) or Barriere or Spuzzum or Sumas or Saanichton or Tsawwassen or Ucluelet or Quadra or Nemaiah Valley or ((Windermeer or Victoria or Vancouver or Terrace or Soda Creek or Surrey or Prince George or Prince Rupert or Parksville or Nelson or Merritt or Langley or Langford or Kimberley or Greenwood or Grand Forks or Duncan or Cranbrook or Courtenay or Colwood or Burnaby or Bonaparte or Armstrong or Abbotsford or Castlegar or Vanderhoof or Valemount or Stewart or Smithers or McBride or Massett or MacKenzie or Houston or Hazelton or Burns Lake or Fraser Lake or Alkali Lake or Ashcroft or Boston Bar or Spences Bridge or Port Douglas or Chase or Litton or Mill Bay or Lomcolith or D'Arcy or Sidney or Agassiz or Harrison Mills or Invermere or Telegraph Creek or Hope or Boothroyd or Trail) and Canad*)).mp.)) or (Duneza or Dunne-za or Dakelh or Babine or Wet'suwet'en or Haida or Sto:lo or Staulo or Stahlo or "Fraser River Indians" or Coast Salish or Kaska or Ktunaxa or Kootenay or Kwakwaka'wakw or Gitxsan or Gwich'in or Gwitich'in or Gitksan or Gwitchin or Gwichin or Kutchin or Tsimshian or Musqueam or St'at'imc or In-SHUCK-ch or Lil'wat or Lillooet or Nisga'a or Nuu-chah-nulth or Nootka or Nuxalk or Sekani or Wuikinuxv or Secwepemc or Sinixt or Skwxwu7mesh or (Tagish not meteorite) or Tahitan or Tahltan or Haisla or ((Nicola or Kitimat or Beaver or Okanagan or Sechelth) adj3 (man or men or boy or boys or girl or girls or adult* or youth* or adolescent* or Nation or people* or Indians* or tribe* or tribal* or band* or bands)) or (Carrier adj3 (Nation or Nations or Indians* or tribe* or tribal*)) or (Tsilhqot'in or Cowichan or Chilcotin or Nlaka'pamux or Tlingit or Tsetsaut or Oweekeno or Kwakiutl or Heiltsuk or Bella Bella or Saulteaux or Bella Coola or Shuswap or quamish or Stl'atl'imx or Stl'atl'imc or Stlatliumh or Slatemuk or Dane-zaa or Tsattine or Tutchone or Tuchone or Akisq'nuk or Esdilagh or Acho Dene Koe or Dene-tha or Adams Lake or Ahousaht or Aitchelitz or Beecher Bay or Blueberry River or Tsleil-Waututh or Burrard or Cacli'p or Canim Lake or Canoe Creek or Dog Creek or Cheam or Chawathil or Aishihi or Chehalis or Chemainus or Cheslatta or Comox or Da'naxda'xw or Ditidaht or Doig River or Dzawada'enuxw or Ehattesaht or Esketemc or Esquimalt or Gitanmaax or Gitanyow or Gitsegukla or Gitwangak or Gitxaala or Glen Vowell or Gwa'sala or Gwawaenuk or Hagwilget or Halalt or Halq'emeylem or Hesquiaht or Homalco or Hupacasath or Hul'quimi'num or Ka:'yu:'k't'h or Che'k'tles7et'h or Katzie or Kispiox or Kitselas or Kitsumlamun or Klahoos or Kluskus or Lhoosk'uz or K'omoks or Kwadacha or Kwaw-kwaw-a-pilt or Kwiakah or Kwicksutaineuk or Kwikwentlem or Lakahahmen or Lax-kw'alaams or Leq'a:mel or Lheidli-T'enneh or Lhatko or Lyackson or Malahat or Mamalilikulla or Matsqui or Metalakatla or Moricetown or Mowachaht or Muchalaht or Musqueam or Nadleh or Nak'azdli or 'Namgis or Sununeymuxw or Nanoos or Nazko or Nee-Tahi-Buhn or Neskonlith or Nisga'a or Nisgaa or Nooaitch or N'quatqua or Nuxalk or Ostlq'emeylem or Pacheedath or Pauquachin or Penelakut or Qayqayt or Quatsino or Saik'uz or Samahquam or Scowlitz or Semiahmoo or Shackan or Shxwha:y or Shw'ow'hamel or Simpcw or (Siska not Karol) or Skatin or Skawahlook or Skin Tyee or Skowkale or Skuppah or Skwah or Sliammon or Soowahilie or Spallumcheen or Squiala or Stellat'en or Taku River or T'it'qet or Tla-o-qui-aht or Tlatlasikwala or Tl'azt'en or Tl'etinqox-t'in or Tlowitsis or Toosey or Toquaht or Tsartlip or Tsawataineuk or Tsawout or Tsay Keh Dene or Tseshaht or Tseycum or Tsi Del Del or Ts'kw'aylaxw or Tsleil-Waututh or T'souke or Tzeachten or Uchcklesaht or Ulkatcho or We Wai Kai or Cape Mudge or Wuikinuxv or Xaxli'p or Yaakweakwioose or Yekooche)).mp.) not (isotope* or radiocarbon* or geology* or stratigraph* or Wisconsin or Michigan or Beaver County or (Alaska not (Alaska and (Canada or British Columbia)))).mp. not (animals not (humans and animals)).sh. [mp=title, book title, abstract, original title, name of substance word, subject heading word, floating sub-heading word, keyword heading word, organism supplementary concept word, protocol supplementary concept word, rare disease supplementary concept word, unique identifier, synonyms, population supplementary concept word, anatomy supplementary concept word]

14 (Opaskawayak or Little Saskatchewan or Fisher River Cree or Peguis or Sagkeeng or Roseau River or Norway House or Sapotaweyak or Wuskwi Siphik or Skownan or Dauphin River or Pinaymootang or Kinonjeoshtegon or O-Chi-Chak-Ko-Sipi or Tootinaowaziibeeng or Lake Manitoba or Keeseekoowenin or Waywayseecappo or Birdtail Sioux or Canupawakpa or Dakota Tipi or Brokenhead Ojibway or Northlands Nursing Station or Lac Brochet or Sayisi Cree or Tadoule Lake or Brochet or O-Pipon-Na-Piwin or South Indian Lake or Mathias Colomb or Pukatawagan or Tataskweyak or York Landing or Nisichawayasihk or Nelson House or Shamattawa or Bunibonibee or Manto Sipi or God's River or God's Lake or Red Sucker Lake or St Theresa Point or Wasagamack or Pauingassi or "Berens River" or Bloodvein).mp. or ((Sandy Bay or Long Plan or Sioux Valley or Fox Lake or War Lake or Pine Creek or Lake St Martin or Hollow Water or Little Black River or Rolling River or Dakota Plains or Swan Lake or Oxford House or Cross Lake or Split Lake or Barren Lands or Garden Hill or Poplar River or Little Grand Rapids).mp. and (exp Canada/ or Canad*.mp.)) or ((exp Indians, North American/ or exp Health Services, Indigenous/ or exp Medicine, Traditional/ or exp Shamanism/ or exp Ethnopharmacology/ or Indigenous*.mp. or Aboriginal*.mp. or Amerindian*.mp. or Autochtone*.mp. or First Nation.mp. or First Nations.mp. or Metis.mp. or (traditional adj1 (medicine* or heal* or food* or health*)).mp. or Urban Indian*.mp. or "on reserve".mp. or "off reserve*".mp. or country food*.mp. or shaman*.mp. or medicine m?n.mp. or medicine wom?n.mp. or ((native* or Indian or Indians) adj2 (person or persons or man or woman or men or women or child* or youth or youths or population* or people* or band or bands)).mp.) and (exp Manitoba/ or Manitoba*.mp. or Winnipeg.mp. or Brandon.mp. or St Boniface.mp. or Dauphin.mp. or Flin Flon.mp. or Morden.mp. or Portage la Prairie.mp. or Selkirk.mp. or Steinbach.mp. or Thompson.mp. or Winkler.mp.)) [mp=title, book title, abstract, original title, name of substance word, subject heading word, floating sub-heading word, keyword heading word, organism supplementary concept word, protocol supplementary concept word, rare disease supplementary concept word, unique identifier, synonyms, population supplementary concept word, anatomy supplementary concept word]

15 ((Ahtahkakoop or Asimakaniseekan or Amisk Lake First Nation* or Assiniboine First Nation* or Beardy or Big River First Nation* or Birch Portage or Bittern Lake or Budd's Point or Mamawetan or Carry the Kettle or Canoe Lake or Carrot River or Chicken First Nation* or Kelsey Trail Health or Sunrise Health or Prince Albert Parkland Health or Day Star or Dipper Rapids or Eagles Lake First Nation* or Elak Dase or English River First Nation* or Prairie North Health or Fishing Lake First Nation* or Flying Dust First Nation* or Fond du Lac or Four Portages or Fox Point First Nation* or Athabasca Health Authority or Gordon First Nation* or Grandmother's Bay or Hatchet Lake or Hay First Nation* or Ils a la Crosse or James Smith First Nation* or Joseph Bighead or Kawacatoose or Kahkewistahaw or Key First Nation* or Kinistin or Kinookimaw or Kitsakie or Knee Lake First Nation* or La Loche First Nation* or La Plonge or Lac La Hache or Lac La Ronge or Little Black Bear or Little Bone First Nation or Little Hills First Nation* or Little Pine First Nation* or Little Red River or Lucky Man or Makaoo or Makaw or Meadow Lake First Nation* or Ministikwan or Minoahchak or Mirond Lake or Mistawasis or Montreal Lake First Nation* or Moosomin First Nation* or Morin Lake or Mosquito-Grizzley Bear's or Muscowpetung or Muskeg Lake First Nation* or Muskeg River First Nation* or Muskoday or Muskowekwan or Nekaneet or New Thunderchild or Cypress Health or Ocean Man or Ochapowace or Okanese or Okemasis or Old Fort First Nation* or One Arrow or Onion Lake or Opawakoscikan or (Pasqua not wheat) or Peepeekisis or Pelican Lake First Nation* or Pelican Narrows First Nation* or Peter Ballantyne or Peter Pond First Nation* or Pheasant Rump or Piapot or Potato River or Poundmaker First Nation* or Primeau Lake First Nation* or Red Earth First Nation* or Red Pheasant or Sakimay or Seekaskootch or Shesheep or Standing Buffalo or Starblanket or Tumor Lake or Wahpeton or Wapachewanak or Wa-Pii or Moos-Toosis or Waterhen or Willow Bunch or Witchekan or Wood Mountain or Yellowquill or Yellow Quill or (northern Saskatchewan not (uranium or selenium))).mp. or ((exp Indians, North American/ or exp Health Services, Indigenous/ or exp Medicine, Traditional/ or exp Shamanism/ or exp Ethnopharmacology/ or (Indigenous* or Aboriginal* or Amerindian* or Autochtone* or First Nation or First Nations or Metis or Michif or Urban Indian* or "on reserve" or "off reserve*" or country food* or residential school* or shaman* or medicine m?n or medicine wom?n or Buffalo River or Island Lake or Lean Man or Pine Bluff or Salteaux or Saulteaux or Sandy Narrows or Shoal Lake or Southend or Stanley or Sturgeon or Sucker River or Sweetgrass or White Bear or White Cap or Woody Lake or Cree or Dene or Chipewyan or Dakota or Algonquian).mp. or (traditional adj1 (medicine* or heal* or food* or health*)).mp. or ((native* or Indian or Indians) adj2 (person or persons or man or woman or men or women or child* or youth or youths or population* or people* or band or bands or reserve or reserves or treaty)).mp.) and (exp Saskatchewan/ or (Saskatchewan* or Sask or Regina or Estevan or Moose Jaw or Saskatoon or Lloydminster or Swift Current).mp.))) not (exp Medicine, Chinese Traditional/ or populus.mp. or India.mp. or Wisconsin.mp. or exp Plant Extracts/ or poplar.mp. or rats.mp. or veterinary.mp. or ve.fs.)

16 ((Aakom Kiyii or Alexis Cardinal River or Alexis Elk River or Alexis Nakota Sioux or Allison Bay or Assineau River or Atikameg or Athabasca Chipweyan or Beaver First Nation or Beaver Lake Cree or Bearspaw or Beaver Ranch or Bigstone Cree or Bistcho Lake or Blood Tribe or Cadotte Lake or Carajou Settlement or Chateh or Chiniki or Chipewyan Prairie or Cold Lake First Nations or Dene Tha or Desmarais Settlement or Dog Head or Driftpile or East Prairie or Elizabeth Settlement or Enilda or Enoch Cree or Duncans First Nation or Ermineskin or Fort Chipewyan or Frog Lake or Garden Creek or Goodfish Lake or Gregoire Lake or Grouard or Heart Lake or Hobbema or Hokedhe or Horse Lake* or Janvier Health or Jere Ghalil or Jean Dor or John Dor or Kainai or Kainaa or Kapaweno or Kee Tas Kee Now or Kehewin or Ki Tue or Kapaweno or Kikino or Kinuso or Jackfish Point or Little Buffalo or Little Red River Cree or Loon Prairie or Louis Bull or Lubicon Lake Indian Nation or Maggie Willier or Makaoo or Maskwacis or Meander Health or Mikisew or Nakota or Nakoda or Namur Lake or OChiese or Old Fort or Paddle Prairie or Paul Band or Paul First Nation or Peace Point or Peavine Settlement or Peigan or Peerless Lake or Peerless Trout or Piikani or Pikuni or Pikani or Puskiakiwenin or Red Earth Creek or Saddle Lake or Samson Cree or Sarcee or Sawridge Band or Siksika or Siksikawa or Sao-kitapiiksi or Stoney First Nation or Stoney Tribe or St Isadore or Sturgeon Lake Cree or Tasttine or Tallcree or Tall Cree or Thabacha or Thebathi or Tsuu Tina or Tsu Tue or Tsu Kadhe or Unipouheos or Utikoomak or Wabasca or Wesley Band or ((Alexander or Athabasca or Beaver Lake or Blue Quill* or Cold Lake or Duncans or Enoch or Fort McKay or Fort McMurray or Loon River or Paul or Smiths Landing or Sucker Creek or Sunchild or Swan River or Whitefish Lake) adj2 (First Nation* or tribe or Indian*))).mp. or (exp Indians, North American/ or exp Health Services, Indigenous/ or exp Medicine, Traditional/ or exp Shamanism/ or exp Ethnopharmacology/ or Indigenous*.mp. or Aboriginal*.mp. or Amerindian*.mp. or Autochtone*.mp. or First Nation.mp. or First Nations.mp. or Dene.mp. or Blackfoot.mp. or Anishinaabe.mp. or Assiniboine.mp. or Metis.mp. or Mischif.mp. or Mitchif.mp. or Metif.mp. or Metchif.mp. or Bois-brule*.mp. or Mixed-blood*.mp. or Half Breed*.mp. or halfbreed*.mp. or (traditional adj1 (medicine* or heal* or food* or health*)).mp. or Urban Indian*.mp. or "on reserve".mp. or "off reserve".mp. or country food*.mp. or shaman*.mp. or medicine m?n.mp. or medicine wom?n.mp. or ((native* or Indian or Indians) adj2 (person or persons or man or woman or men or women or child* or youth or youths or population* or people* or band or bands)).mp.)) and (Beaver Lake or Brownvale or Fort McMurray or Edmonton or Calgary or Hythe or Slave Lake or Valleyview or Fort Vermilion or Morinville or Glenevis or Lac La Biche or Cold Lake or Rocky Mountain House or Duffield or Brocket or Morley or Whitecourt or Amber River or Big Horn or Buck Lake or Charles Lake or Collin Lake or Cornwall Lake or Cowper Lake or Devils Gate or Eden Valley or Fox Lake or Little Red River or Hay Lake or Lesser Slave Lake or Bonneyville or Loon Lake or Wetaskiwin or Pigeon Lake or Lake Athabasca or Fort McLeod or Barrhead or Stony Plain or Sturgeon Lake or High Prairie or Swampy Lake or Upper Hay River or Wabamun or Trout Lake or Whitefish Lake or Winefred Lake or Nordegg or Boyer River or Calling Lake or Berwyn or Fort Chipewyan or Black Diamond or Fishing Lake or Gift Lake or Kananaskis or Medicine Hat or Ponoka or Stand Off or StandOff or Alberta).mp.

17 (exp Indians, North American/ or exp Inuits/ or exp Health Services, Indigenous/ or exp Shamanism/ or exp Medicine, Traditional/ or exp Ethnopharmacology/ or American Native Continental Ancestry Group/ or (Abenaki or Abenakis or Abitibiwinni or Akwesasne* or Atikamek* or "Barriere Lake" or Betsiamite* or Cacouna or Chisasibi or Coucoucache or "Eagle Village" or Eastmain or Essipit* or Ekuanitshit* or Gespeg or Gesgapegiag* or Huron-Wendat or "Huronne Wendat" or Essipit or Inuit or Inuk or Kahnawake or Kahnawa?ke or Kanesatake or Kawawachikamach* or Kebaowek or Kipawa or Kitcisakik or Kitigan Zibi or Kuujjuaq or "Lac Romanie" or "Lac John" or "Lac Simon" or Listuguj* or "Long Point First" or Maliotenam or Maliseet or Malecite* or Mamit Innuat or Mamuitun or Manawan or Mashteuiatsh or Matimekush* or Matimekosh* or Mawiomi or Migmaw or Mig Maw or Mi?gmawei or Mingan or Mistissini* or Montagnais or Naskapi* or Natashquan* or Nemiscau or Nemaska* or Obedjiwan or Odanak or Opitciwa* or dopitciwa* or Ouje?Bougoumou or Pakuashipi* or Pessamit* or Pikogan or "Rapid Lake" or Salluit or Schefferville or Sept-Iles or Takuaikan or "Uashat Mak" or "Mani-Utenam" or Temiscaming or imiskaming or Ungava or Uashat or "Unamen Shipu" or Waban-Aki or Waskaganish or Waswanipi or Wemindji or Wemotaci or Wendat* or Wendake or Whapmagoostui or Wolinak or Kitcisakik or "Pakua Shipu" or "Pakua Shipi" or Winneway or (Peuple* adj (autochtone* or indigene* or premier* or racine* or natif*)) or "Premiere Nation" or "First Nation" or "First Nations" or Metis or Cree or Algonquin* or Algonquian* or Anishinabe* or Anishinaabeg* or autochton* or Inuit* or Innu or Innus or Innue or Micmac or Mic?Mac or Mi?gmaq or Mi?kmaq or Mowhawk or (urban adj3 (Indian* or Native* or Aboriginal*)) or (Native adj3 (american or man or men or women or woman or boy* or girl* or children or people* or indian* or Nation or tribe* or tribal or band or bands or groups or communit* or population* or health)) or indigenous* or Aboriginal* or autochtone* or treaty or on-reserve or "off reserve" or country food* or Shaman* or (traditional adj (medicine or heal*)) or residential school*).mp.) and (exp Quebec/ or (montreal or "trois rivieres" or quebec* or "james bay" or "baie james" or Laval or Gatineau or Longueuil or Sherbrooke or Saguenay or Doncaster or Levis or Terrebonne or Mascouche or l?Estrie* or Lanaudiere or l?Outaouais* or Capitale-Nationale or Chaudiere-Appalaches or Cote-Nord or Gaspe* or Mauricie or Monteregie or Laurentides or Bas-Saint-Laurent or Saint-Laurent or Nunavik).mp. or (QC or quebec).in.)

18 ((Aamjiwnaang or Pikwakanagan or (Animbiigoo adj Zaagi?igan adj Anishinaabek*) or Wauzhushk Onigum or Naongashiing or Anishnabekwe or Anishnawbe* or "ARMSTRONG SETTLEMENT" or Aroland or ASSABASKA or "Atikameksheng Anishnawbek" or Attawapiskat or "Aundeck-Omni-Kaning" or Batchewana or "Bearfoot Onondaga" or "Biinjitiwabik Zaaging" or Bimose or "Bingwi Neyaashi" or Bkejwanong or "chapleau cree" or dokis or Eabametoong or "Fox Lake Cree" or (moravian adj2 thames) or Ginoogaming or "Kasabonika Lake" or Kashechewan or KABAPIKOTAWANGAG or Keewaytinook or Kee?Way?Win or "Kiashke Zaaging" or Kitchenuhmaykoosib or Konadaha Seneca or Koocheching or Magnetawan or Matachewan or Mattagami or "MacDowell Lake" or M?Chigeeng or Mishkeegogamang or Missanabie or Mitaanjigaming or Stanjikoming or Mocreebec or (Mohawks adj (Akwesasne or Gibson)) or "Moose Cree" or Naicatchewenin or Namaygoosisagagun or Naotkamegwanning or Neskantaga or Nibinamik or Nigigoonsiminikaaning or Nipissing or OBADJIWAN or Obashkaandagaang or "Washagamis Bay" or Ochiichagwe Bibigo?ining or Onigaming or Parmachene or Sabaskong or Sagamok or (Oneida Nation adj2 Thames) or Shawanaga or Sheguiandah or Sheshegwaning or Taykwa Tagamou or Temagami or Wabaseemoong or Wabauskang or "Wabigoon Lake" or Wahgoshig or Wahnapitae or "Wahta Mohawks" or Wapekeka or Wasauksing or Wauzhushk Onigum or Wawakepewin or Webequie or Weenusk or Wikwemikong or ((Ardoch or Algonquin or Beausoleil or "big grassy" or "Iskatewizaagegan 39 Independent" or beaverhouse or "brunswick house" or "buffalo point" or caldwell or "cat lake" or Couchiching or "Curve Lake" or "deer lake" or delaware or "duck lake" or "Eagle Lake" or Thames or "Fort William" or "Garden River" or "Grassy Narrows" or Hiawatha or Henvey Inlet or Hornepayne or "Gull Bay" or "King Fisher" or (Lac adj Mille adj Lacs) or (Lac adj Croix) or "Long Lake" or Magnetawan or "Marten Falls" or "Martin Falls" or Mississauga or "New Credit" or "Moose Deer Point" or Munsee?Delaware or "Muskrat Dam" or "North Caribou Lake" or "North Spirit Lake" or Northwest Angle or Sioux Narrows or Pays Plat or Pic Mobert or "Red Rock" or "Sachigo Lake" or "Sandy Lake" or "Savant Lake" or Saugeen or Seine River or Serpent River or "Shoal Lake" or Stony Point or Stoney Point or "Grand River Territory" or "Slate Falls" or Whitefish River or Whitesand or "Whitewater Lake" or "Wunnumin Lake") adj2 first nation*)).mp. or (exp Indians, North American/ or exp Inuits/ or exp Health Services, Indigenous/ or exp Shamanism/ or exp Medicine, Traditional/ or exp Ethnopharmacology/ or American Native Continental Ancestry Group/ or (Peuple adj (autochtones or indidgenes or premier or racing or natif*)).mp. or ("Premiere Nation" or "First Nation" or "First Nations" or Metis or chippewas or Cree or Algonquin* or Algonquian* or Anishinabe* or Anishnabeg or Anishinaabe* or autochthon* or Inuit* or Innu or Innus or Innue or Micmac or Mic?Mac or Mi?gmaq or Mi?kmaq or Mowhawk or Ojibw* or Cayuga).mp. or (urban adj3 (Indian* or Native* or Aboriginal*)).mp. or ((Native not (bacteri* or plant* or species or micro* or biot* or strain or strains or probiotic or zoo* or geno*)) adj3 (american or man or men or women or woman or boy* or girl* or children or people* or indian* or Nation or tribe* or tribal or band or bands or groups or communit* or population* or health)).mp. or ((indigenous* not (bacteri* or plant* or species or micro* or biot* or strain or strains or probiotic or zoo* or geno*)) or Aboriginal* or (autochon* not (bacteri* or plant* or species or micro* or biot* or strain or strains or probiotic or zoo* or geno*)) or treaty or on-reserve or off-reserve or country food* or Shaman* or (traditional adj (medicine or heal*)) or residential school*).mp.)) and (exp Ontario/ or (ontario or toronto or ottawa or sarnia or london or hamilton or windsor or roseneath or "golden lake" or beardmore or "christian island" or "Cedar Point" or morson or "bay of quinte" or "bear island" or "Bearskin Lake" or "whitefish lake" or naughton or "little current" or "Kirkland lake" or "big island" or macdiarmid or "thunder bay" or wallaceburg or chapleau or leamington or "georgina island" or "CAPE CROKER" or "FOX LAKE" or "CHIEF’S POINT" or "Constance Lake" or "Curve Lake" or "deer lake" or "duck lake" or "Eagle Lake" or "ENGLISH RIVER" or Rama or Erie St?Clair or "FACTORY ISLAND" or "Flying Post" or "FORT ALBANY" or "FORT HOPE" or "Fort Severn" or "Fort William" or Wiarton or Southampton or Saugeen or Muncy or Calstock or "Fort Frances" or "FRENCH RIVER" or Thamesville or Monteville or "Eabamet Lake" or Nipigon or "Georgian Bay" or "Long Lac" or "Long Lake" or Grassy Narrows or Keene or Pickerel or Hornepayne or Glebe Farm or "GOULAIS BAY" or Grey? Owen Sound or GROS CAP or "Shoal Lake" or Kashechewan or INDIAN RIVER or "King Fisher Lake" or "Big Trout Lake" or "Sandy Lake" or "Fort Frances" or "Lac Seul" or "LAKE HELEN" or (LAKE adj WOODS) or Hudson or Britt or Okoki Post or "MANITOU RAPIDS" or gogama or "McDowell Lake" or "Red Lake" or Wawa or "New Osnaburgh" or "Garden River" or "Blind River" or Hagersville or "Port Perry" or "Moose Factory" or Cornwall or Deseronto or Mactier or Muncey or Delvin or Pawitik or "Whitefish Bay" or "Landsdowne House" or "Summer Beaver" or "Weagamow Lake" or "North Spirit Lake" or "Rainy Lake" or "Rainy River" or "Sioux Narrows" or Keewating or Kenora or "Sault Ste Marie" or "Nestor Falls" or "Heron Bay" or Southwold or "Pays Plat" or Mobert or "Pic River" or "Moon River" or Massey or "Sachigo Lake" or "Savant Lake" or Cutler or Nobel or Sheshegwaning or Kejick or Ohsweken or "Slate Falls" or Cochrane or "Lake Tamagami" or Thessalon or Whitedog or "Ear Falls" or Dryden or Matheson or Bala or Capreol or "Angling Lake" or "Parry Sound" or "Sioux Lookout" or Webequie or Peawanuk or "Birch Island" or Armstrong or "Wunnumin Lake" or Silverwater or Walpole Island or WINISK).mp.)

19 ((Carcross or (Tagish not meteorite*) or Champagne First Nation or Aishihik or Ehdiitat or Nacho Nyak Dun or Gwichya or Little Salmon or Carmacks or Nihtat or Selkirk First Nation or Ta'an Kwach'an or Tetlitn or Tr'ondek Hwech'in or White River First Nation or Vuntut or Yellowknives or (Hare adj2 (man or men or woman or women or child* or youth* or adult* or people* or person or persons or tribe or tribal or band or bands)) or Tanana or Tanana or Tutchone* or Denesuline or Tahltan or MacKenzie Valley or Old Crow or "Upper Liard" or "Eagle Plains" or "Keno City" or Carcross or Teslin or "Fort Selkirk" or Carmacks or Haines Junction or Dawson City).mp. or ((Canad*.mp. or exp Canada/) and (Beaver Creek or Pelly or Destruction Bay or Watson Lake).mp.) or ((exp Indians, North American/ or exp Health Services, Indigenous/ or exp Medicine, Traditional/ or exp Shamanism/ or exp Ethnopharmacology/ or Indigenous*.mp. or Aboriginal*.mp. or Amerindian*.mp. or Autochtone*.mp. or Metis.mp. or First Nation.mp. or First Nations.mp. or exp Inuit/ or Inuit*.mp. or Chipewyan.mp. or Kaska.mp. or Kaskas.mp. or Tlingit.mp. or Dene.mp. or Gwich'in.mp. or Gwichin.mp. or Gwitchin.mp. or Kutchin*.mp. or Sahtu.mp. or Tlicho.mp. or Tli Cho.mp. or (traditional adj1 (medicine* or heal* or food* or health*)).mp. or Urban Indian*.mp. or "on reserve".mp. or "off reserve*or country food*".mp. or shaman*.mp. or medicine m?n.mp. or medicine wom?n.mp. or treaty.mp. or treaties.mp. or ((native* or Indian or Indians) adj2 (person or persons or man or woman or men or women or child* or youth or youths or population* or people* or band or bands)).mp.) and (exp Yukon Territory/ or Yukon*.mp. or ((Beaufort Sea or Whitehorse) and Canad*).mp.))) not ((exp Alaska/ or Alaska*.mp.) not ((exp Alaska/ or Alaska*.mp.) and (exp Yukon/ or Yukon*.mp.))) not (Yukon-Kuskok* or lepus or geology* or stratigraphi* or subduction* or volcan* or Holocene or pleistocene).mp. [mp=title, book title, abstract, original title, name of substance word, subject heading word, floating sub-heading word, keyword heading word, organism supplementary concept word, protocol supplementary concept word, rare disease supplementary concept word, unique identifier, synonyms, population supplementary concept word, anatomy supplementary concept word]

20 ((((exp Medicine, Traditional/ not Chinese.mp.) or exp Shamanism/ or exp Indians, North American/ or exp Inuits/ or exp Health Services, Indigenous/ or exp Ethnopharmacology/ or (Inuit* or Eskimo* or Esquimau* or Athapaskan or Gwich'in or Metis or Inuvialuktun or Cree or Aboriginal* or Indigenous* or off-reserve or on-reserve or First Nation or First Nations or Amerindian or (urban adj3 (Indian* or Native* or Aboriginal*)) or ethnomedicine or country food* or residential school* or shaman* or traditional medicine* or traditional heal* or traditional food* or medicine man or medicine woman or autochtone* or treaty or (Native adj1 (man or men or women or woman or boy* or girl* or adolescent* or youth or youths or person* or adult or people* or Indian* or Nation or tribe* or tribal or band or bands))).mp.) and (exp Northwest Territories/ or Northwest Territories.mp. or NWT.mp. or Yellowknife.mp. or Western Arctic.mp.)) or (Aklavik or Banks Island or Behchoko or Rae Edzo or Colville Lake or De Cho or Deline or Denendeh or Fort Good Hope or Fort Liard or Fort McPherson or Fort McPherson or Fort Providence or Fort Providence or Fort Simpson or Fort Smith or Gameti or Hay River or Inuvik or Jean Marie River or Lutselk'e or Norman Wells or Paulatuk or Sachs Harbour or Trout Lake or Tsiigehtchic or Tuktoyaktuk or Tulita or Tulit'a or Ulukhaktok or Victoria Island or Whati or Wha Ti or Wrigley or (Hare adj2 (man or men or boy or boys or girl or girls or adult* or youth* or adolescent* or Nation or people* or Indians* or tribe* or tribal*)) or Slavey or Chipewyan or Tlicho or Dogrib or Yellowknives or Dene or Sahtu or Inuvaluit* or Inuinnaqtun).mp.) not ((fort smith adj1 ar*).mp. or ((rabbit* or lepus or lemming* or fox or foxes or wolf or wolves or (wrigley adj1 (n or g or forcep*))).mp. or ve.fs.)) [mp=title, book title, abstract, original title, name of substance word, subject heading word, floating sub-heading word, keyword heading word, organism supplementary concept word, protocol supplementary concept word, rare disease supplementary concept word, unique identifier, synonyms, population supplementary concept word, anatomy supplementary concept word]

21 (exp Nunavut/ or Nunavut.mp. or Eastern Arctic.mp. or Alert Bay.mp. or Alexandra Fiord.mp. or Amadjuak.mp. or Aquiatulavik Point.mp. or Arctic Bay.mp. or Arviat.mp. or Baffin Island.mp. or Baker Lake.mp. or Bathurst Inlet.mp. or Belcher Islands.mp. or Bylot Island.mp. or Cambridge Bay.mp. or Iqaluktuttiaq.mp. or Cape Dorset.mp. or Cape Dyer.mp. or Cape Smith.mp. or Charlton Depot.mp. or Chesterfield Inlet.mp. or Clyde River.mp. or Coral Harbour.mp. or Craig Harbour.mp. or Dundas Harbor.mp. or Ellesmere Island.mp. or Ennadai.mp. or Eskimo Point.mp. or Fort Conger.mp. or Fort Hope.mp. or Fort Ross.mp. or Gjoa Haven.mp. or Grise Fiord.mp. or Hall Beach.mp. or Hazen Camp.mp. or Igloolik.mp. or Ikaluit.mp. or Iqaluit.mp. or Isachsen.mp. or Kekerten.mp. or Kimmirut.mp. or King William Island.mp. or Kipisa.mp. or Kitikmeot o r Kivalliq.mp. or Kivitoo.mp. or Kugaaruk.mp. or Kugluktuk.mp. or Maguse River.mp. or Nanasivik.mp. or Nottingham Island.mp. or Nuwata.mp. or Padlei.mp. or Padloping Island.mp. or Pangnirtung.mp. or Perry Island.mp. or Pond Inlet.mp. or Port Burwell.mp. or Qoloqtaaluk.mp. or Qikiqtarjuaq.mp. or Rankin Inlet.mp. or Read Island.mp. or Repuilse Bay.mp. or Resolute Bay.mp. or Resolution Island.mp. or Sanikiluak.mp. or Taloyoak.mp. or Tanquary Camp.mp. or Tavani.mp. or Thom Bay.mp. or Umingmaktok.mp. or Victoria Island.mp. or Wager Bay.mp. or Whale Cove.mp. or Eastern Arctic.mp. or ((Lupin or Polaris or Eureka or Fullerton) and Canad*).mp.) not (exp behavior, animal/ or exp ecosystems/ or exp endangered species/ or (sediment* or mantle or basalt* or cretaceous* or fossil* or paleo* or geolog* or stratigraph* or glaci* or refugia* or moraine* or pliocene or gravity or methylmercury or hydrolog* or hydrogeol* or volcan* or mesospher* or inferomet* or habitat* or animal behavior* or endangered species).mp.) [mp=title, book title, abstract, original title, name of substance word, subject heading word, floating sub-heading word, keyword heading word, organism supplementary concept word, protocol supplementary concept word, rare disease supplementary concept word, unique identifier, synonyms, population supplementary concept word, anatomy supplementary concept word]

22 (exp Indians, North American/ or exp Health Services, Indigenous/ or Metis.mp. or exp Medicine, Traditional/ or exp Shamanism/ or exp Ethnopharmacology/ or Indigenous*.mp. or Aboriginal*.mp. or Amerindian*.mp. or Autochtone*.mp. or Metis.mp. or First Nations.mp. or First Nation*.mp. or (traditional adj1 (medicine* or heal* or food* or health*)).mp. or Urban Indian*.mp. or "on reserve".mp. or "off reserve*".mp. or country food*.mp. or residential school*.mp. or shaman*.mp. or medicine m?n.mp. or medicine wom?n.mp. or ((native* or Indian or Indians) adj2 (person or persons or man or woman or men or women or child* or youth or youths or population* or people* or band or bands)).mp. or Montagnais.mp. or Maliseet.mp. or Naskapi*.mp. or Mi'kmaq.mp. or Micmac.mp. or Mic mac.mp. or Migmaw.mp. or Mig maw.mp. or Beothuk*.mp.) and (((Fredrickton or Moncton or New Jersey).mp. and (exp Canada/ or Canad*.mp.)) or exp New Brunswick/ or (New Brunswick* not ("New Brunswick NJ" or New Jersey or ferment*)).mp. or Nouveau Brunswick.mp. or Big Hole Tract.mp. or Metepenagiag.mp. or Eel Ground First Nation.mp. or Buctouche.mp. or Esgenoopetitj.mp. or Burnt Church.mp. or Devon Reserve.mp. or St Mary's First Nation.mp. or Eel River Reserve.mp. or Eel River Bar.mp. or Fort Folly Indian Point Reserve.mp. or Indian Island First Nation.mp. or Indian Ranch Reserve.mp. or Kingsclear.mp. or St John River Valley Tribal Council.mp. or Oromocto.mp. or Pabineau.mp. or Pokemouche.mp. or Mawiw.mp. or (Red Bank adj2 Reserve).mp. or Richibucto.mp. or St Basile.mp. or Madawaska.mp. or Soegao.mp. or Tabusintac.mp. or Tobique.mp. or Wolastoqiyik.mp. or Woodstock First Nation.mp.) [mp=title, book title, abstract, original title, name of substance word, subject heading word, floating sub-heading word, keyword heading word, organism supplementary concept word, protocol supplementary concept word, rare disease supplementary concept word, unique identifier, synonyms, population supplementary concept word, anatomy supplementary concept word]

23 (((((Indigenous* or Aboriginal* or Amerindian* or Autochtone* or First Nation or First Nations or (traditional adj1 (medicine* or heal* or food* or health*)) or Urban Indian* or "on reserve" or "off reserve*" or country food* or shaman* or medicine m?n or medicine wom?n or (native* or Indian or Indians)) adj2 (person or persons or man or woman or men or women or child* or youth or youths or population* or people* or band or bands)) or Montagnais or Maliseet or Naskapi* or Mi'kmaq or Micmac or Mic mac or Migmaw or Mig maw or Beothuk*).mp. or exp Health Services, Indigenous/ or exp Indians, North American/ or Metis.mp. or exp Medicine, Traditional/ or exp Shamanism/ or exp Ethnopharmacology/) and (((Wolfville or Middleton or Kentville or Berwick or Inverness or New Waterford or Sackville or Springhill or Halifax or Dartmouth or Truro or New Glasgow or Sydney or Canso or Guysborough or Parrsboro or Pictou or Liverpool or Lunenburg or Amherst) and Canad*).mp. or exp Nova Scotia/ or Nova Scotia*.mp. or Nouvelle Ecosse.mp. or Pictou Landing.mp. or Bear River.mp. or Boat Harbour.mp. or Annapolis Royal.mp. or Antigonish.mp. or Baddeck.mp. or Cheticamp.mp. or Cape Breton.mp. or Neil's Harbour.mp. or Glace Bay.mp. or Tatamagouche.mp. or Sheet Harbour.mp. or Cambridge Reserve.mp. or Annapolis Valley First Nation.mp. or Chapel Island First Nation.mp. or Cole Harbour.mp. or Eskasoni.mp. or Fisher's Grant.mp. or Franklin Manor.mp. or Paq'tnkek.mp. or (Glooscap adj1 (First Nation or reserve)).mp. or Acadia First Nation.mp. or Gold River Reserve.mp. or Horton Reserve.mp. or Shubenacadie First Nation.mp. or Indian Brook Reserve.mp. or Wagmatcook.mp. or Waycobah.mp. or Millbrook First Nation.mp. or Malagawatch.mp. or Medway River.mp. or Membertou.mp. or Merigomish.mp. or Musquodoboit.mp. or New Ross Reserve.mp. or Pennal Reserve.mp. or Pomquet.mp. or Poonhook.mp. or Sheet Harbour.mp. or St Croix Reserve.mp. or Summerside Reserve.mp. or Sydney Reserve.mp. or Truro Reserve.mp. or We'koqma'q.mp. or Wycocomagh.mp. or Wildcat Reserve.mp. or Yarmouth Reserve.mp.)) not (geology or geologic or stratigraphy* or animal* or cat or cats or kitten or deer or bird* or dog or dogs or feline or canine or bovine or equine or porcine or pig or piglet or swine or rat or rats or horse or horses or mouse or mice).mp. [mp=title, book title, abstract, original title, name of substance word, subject heading word, floating sub-heading word, keyword heading word, organism supplementary concept word, protocol supplementary concept word, rare disease supplementary concept word, unique identifier, synonyms, population supplementary concept word, anatomy supplementary concept word]

24 ((exp Indians, North American/ and Canad*.mp.) or Indigenous Canadians/ or exp Inuits/ or exp Health Services, Indigenous/ or exp Ethnopharmacology/ or (Athapaskan or Saulteaux or Wakashan or Cree or Dene or Inuit or Inuk or Inuvialuit* or Haida or Ktunaxa or Tsimshian or Gitxsan or Gitksan or "Nisga'a" or Haisla or Heiltsuk or Oweenkeno or "Kwakwaka'wakw" or "Nuu chah nulth" or "Tsilhqot'in" or Dakelh or "Wet'suwet'en" or Sekani or Dunne-za or Dene or Tahltan or Kaska or Tagish or Tutchone or Nuxalk or Salish or St'at'imc or Stl'atl'imx or Stl'atl'imc or Nlaka'pamux or Okanagan or "Sec wepmc" or Secwepemc or Tlingit or Anishinaabe or Blackfoot or Nakoda or Tasttine or "Tsuu T'ina" or "Tsuut'ina" or "Gwich'in" or Han or Algonquin or Nipissing or Ojibwa or Potawatomi or Innu or Maliseet or "Mi'kmaq" or Micmac or Passamaquoddy or Haudenosaunee or Cayuga or Mohawk or Oneida or Onondaga or Seneca or Tuscarora or Wyandot or Aboriginal* or Indigenous* or Metis or red road or "on reserve" or off-reserve or First Nation or First Nations or Amerindian).mp. or (urban adj3 (Indian* or Native* or Aboriginal*)).mp. or ethnomedicine.mp. or country food*.mp. or residential school*.mp. or ((exp Medicine, Traditional/ or traditional medicine*.mp.) not Chinese.mp.) or exp Shamanism/ or shaman*.mp. or traditional heal*.mp. or traditional food*.mp. or medicine man.mp. or medicine woman.mp. or autochtone*.mp. or (Native* adj1 (man or men or women or woman or boy* or girl* or adolescent* or youth or youths or person* or adult or people* or Indian* or Nation or tribe* or tribal or band or bands)).mp.) and (exp Canada/ or (Canad* or British Columbia or Colombie Britannique or Alberta or Saskatchewan or Manitoba or Ontario or Quebec or Nova Scotia or New Brunswick or Newfoundland or Labrador or Prince Edward Island or Yukon Territory or NWT or Northwest Territories or Nunavut or Nunavik or Nunatsiavut or NunatuKavut).mp.) [mp=title, book title, abstract, original title, name of substance word, subject heading word, floating sub-heading word, keyword heading word, organism supplementary concept word, protocol supplementary concept word, rare disease supplementary concept word, unique identifier, synonyms, population supplementary concept word, anatomy supplementary concept word]

25 12 or 13 or 14 or 15 or 16 or 17 or 18 or 19 or 20 or 21 or 22 or 23 or 24

26 Disability Evaluation/

27 Disabled Persons/

28 "Activities of Daily Living"/

29 "Quality of Life"/

30 Functional Status/

31 Mobility Limitation/

32 Physical Functional Performance/

33 exp "International Classification of Functioning, Disability and Health"/

34 (disabilit* or disabl*).ti,ab,kw.

35 impair*.ti,ab,kw.

36 (functioning* or functional*).ti,ab,kw.

37 (activit* adj2 (limit* or restrict*)).ti,ab,kw.

38 (participat* adj2 (limit* or retrict*)).ti,ab,kw.

39 (function* adj2 (outcome* or limit* or restrict*)).ti,ab,kw.

40 (mobility* adj3 (restrict* or limit* or loss* or lose* or losing* or lost*)).ti,ab,kw.

41 (restrict* adj2 back).ti,ab,kw.

42 (HRQOL* or HAQ or Health Assessment Questionnaire).ti,ab,kw.

43 (PROMIS or Patient Reported Outcomes Measurement Information System).ti,ab,kw.

44 (PROM or PROMS or patient reported outcome measure).ti,ab,kw.

45 (PSFS or Patient Specific Functional Scale).ti,ab,kw.

46 oswestry*.ti,ab,kw.

47 Roland-Morris*.ti,ab,kw.

48 (WHO-DAS* or WHODAS* or WHO DAS).ti,ab,kw.

49 Pain Disability Index.ti,ab,kw.

50 26 or 27 or 28 or 29 or 30 or 31 or 32 or 33 or 34 or 35 or 36 or 37 or 38 or 39 or 40 or 41 or 42 or 43 or 44 or 45 or 46 or 47 or 48 or 49

51 Chronic Pain/

52 exp Pain/

53 exp Neuralgia/

54 Sciatica/

55 Pain Management/

56 Pain Clinics/

57 Pain Measurement/

58 (pain* or allodyn* or analges* or anesth* or anaesth* or causalg* or dysesthes* or hyperalg* or hyperesthes* or hyperpath* or hypoalges* or hypoesthes* or neuralg* or ache or aches or headache* metatarsalg* or arthralg* or neuritis* or neuropath* or nocicept* or "noxious stimulus" or paresthes* or sensitization* or emotion* or mental* or psycholog* or physical* or intergenerat* or spirit*).ti,ab,kw.

59 51 or 52 or 53 or 54 or 55 or 56 or 57 or 58

60 25 and (50 or 59)

61 (comment or editorial or letter or review or systematic review or guideline or practice guideline or case reports or randomized controlled trial).pt.

62 exp Animals/

63 exp Humans/

64 62 not 63

65 60 not (61 or 64)

Database(s): **Ovid MEDLINE(R) and Epub Ahead of Print, In-Process, In-Data-Review & Other Non-Indexed Citations, Daily and Versions**1946 to February 07, 2024

| **#** | **Searches** | **Results** |
| --- | --- | --- |
| 1 | Indigenous Peoples/ | 1427 |
| 2 | Health Services, Indigenous/ | 4229 |
| 3 | Indians, North American/ | 15151 |
| 4 | Inuits/ | 4075 |
| 5 | Indigenous Canadians/ | 130 |
| 6 | (indigenous* or aboriginal* or "first nations" or "first nation" or inuit* or inuk* or metis* or michif* or "Native people" or "native peoples").mp. | 60374 |
| 7 | exp Canada/ | 184933 |
| 8 | ((turtle* adj island*) or canad* or nunavut* or yukon* or (northwest adj1 territor*) or (british adj1 columbia*) or alberta* or saskatchewan* or manitoba* or ontario* or quebec* or new brunswick or prince edward island or nova scotia or newfoundland or toronto* or vancouver* or montreal* or ottawa* or calgary* or edmonton* or winnipeg* or regina* or hamilton* or halifax*).mp. | 346848 |
| 9 | 1 or 2 or 3 or 4 or 6 | 71761 |
| 10 | 7 or 8 | 346848 |
| 11 | 9 and 10 | 8521 |
| 12 | 5 or 11 | 8521 |
| 13 | (((exp Indians, North American/ or exp Inuits/ or exp Health Services, Indigenous/ or exp Ethnopharmacology/ or (exp Medicine, Traditional/ not Chinese.mp.) or exp Shamanism/ or (Athapaskan or Saulteaux or Wakashan or Cree or Aboriginal* or Indigenous* or Metis or "off-reserve" or "on reserve" or First Nation or First Nations or Amerindian or (urban adj3 (Indian* or Native* or Aboriginal*)) or ethnomedicine or country food* or residential school* or shaman* or traditional medicine* or traditional heal* or traditional food* or medicine man or medicine woman or autochtone* or (Native adj1 (man or men or women or woman or boy* or girl* or adolescent* or youth or youths or person* or adult or people* or Indian* or Nation or tribe* or tribal or band or bands))).mp.) and (exp British Columbia/ or (British Columbia or Colombie Britannique or Williams Lake or Vernon or White Rock or Salmon Arm or Quesnel or Powell River or Port Moody or Port Hardy or Port Coquitlam or Port Albernie or Pitt Meadows or Penticton or New Westminister or Nanaimo or Kelowna or Kamloops or Fort St John or Fernie or Enderby or Dawson Creek or Coquitlam or Chilliwack or Campbell River or Tumbler Ridge or Skidegate or Sandspit or Queen Charlotte or Port Clements or Kitimat or Hudsons Hope or Haida Gwaii or Fort St James or Fort Nelson or Dease Lake or Pouce Coupe or Chetwynd or Gransile or Atlin or Alexis Creek or Sooke or Cache Creek or Chehalis or Cheslatta or Kingcome Inlet or Kitwanga or Iskut or Kyuqot or Kanaka or Clo-oose or Nicomen or Liard River or Mount Currie or Keremos or Matsqui or Nanaimo or Tahsis or Gitwinkshilkw or Osoyoos or Popkum or Sechelt or Skookumchuk or (Siska not Karol) or Barriere or Spuzzum or Sumas or Saanichton or Tsawwassen or Ucluelet or Quadra or Nemaiah Valley or ((Windermeer or Victoria or Vancouver or Terrace or Soda Creek or Surrey or Prince George or Prince Rupert or Parksville or Nelson or Merritt or Langley or Langford or Kimberley or Greenwood or Grand Forks or Duncan or Cranbrook or Courtenay or Colwood or Burnaby or Bonaparte or Armstrong or Abbotsford or Castlegar or Vanderhoof or Valemount or Stewart or Smithers or McBride or Massett or MacKenzie or Houston or Hazelton or Burns Lake or Fraser Lake or Alkali Lake or Ashcroft or Boston Bar or Spences Bridge or Port Douglas or Chase or Litton or Mill Bay or Lomcolith or D'Arcy or Sidney or Agassiz or Harrison Mills or Invermere or Telegraph Creek or Hope or Boothroyd or Trail) and Canad*)).mp.)) or (Duneza or Dunne-za or Dakelh or Babine or Wet'suwet'en or Haida or Sto:lo or Staulo or Stahlo or "Fraser River Indians" or Coast Salish or Kaska or Ktunaxa or Kootenay or Kwakwaka'wakw or Gitxsan or Gwich'in or Gwitich'in or Gitksan or Gwitchin or Gwichin or Kutchin or Tsimshian or Musqueam or St'at'imc or In-SHUCK-ch or Lil'wat or Lillooet or Nisga'a or Nuu-chah-nulth or Nootka or Nuxalk or Sekani or Wuikinuxv or Secwepemc or Sinixt or Skwxwu7mesh or (Tagish not meteorite) or Tahitan or Tahltan or Haisla or ((Nicola or Kitimat or Beaver or Okanagan or Sechelth) adj3 (man or men or boy or boys or girl or girls or adult* or youth* or adolescent* or Nation or people* or Indians* or tribe* or tribal* or band* or bands)) or (Carrier adj3 (Nation or Nations or Indians* or tribe* or tribal*)) or (Tsilhqot'in or Cowichan or Chilcotin or Nlaka'pamux or Tlingit or Tsetsaut or Oweekeno or Kwakiutl or Heiltsuk or Bella Bella or Saulteaux or Bella Coola or Shuswap or quamish or Stl'atl'imx or Stl'atl'imc or Stlatliumh or Slatemuk or Dane-zaa or Tsattine or Tutchone or Tuchone or Akisq'nuk or Esdilagh or Acho Dene Koe or Dene-tha or Adams Lake or Ahousaht or Aitchelitz or Beecher Bay or Blueberry River or Tsleil-Waututh or Burrard or Cacli'p or Canim Lake or Canoe Creek or Dog Creek or Cheam or Chawathil or Aishihi or Chehalis or Chemainus or Cheslatta or Comox or Da'naxda'xw or Ditidaht or Doig River or Dzawada'enuxw or Ehattesaht or Esketemc or Esquimalt or Gitanmaax or Gitanyow or Gitsegukla or Gitwangak or Gitxaala or Glen Vowell or Gwa'sala or Gwawaenuk or Hagwilget or Halalt or Halq'emeylem or Hesquiaht or Homalco or Hupacasath or Hul'quimi'num or Ka:'yu:'k't'h or Che'k'tles7et'h or Katzie or Kispiox or Kitselas or Kitsumlamun or Klahoos or Kluskus or Lhoosk'uz or K'omoks or Kwadacha or Kwaw-kwaw-a-pilt or Kwiakah or Kwicksutaineuk or Kwikwentlem or Lakahahmen or Lax-kw'alaams or Leq'a:mel or Lheidli-T'enneh or Lhatko or Lyackson or Malahat or Mamalilikulla or Matsqui or Metalakatla or Moricetown or Mowachaht or Muchalaht or Musqueam or Nadleh or Nak'azdli or 'Namgis or Sununeymuxw or Nanoos or Nazko or Nee-Tahi-Buhn or Neskonlith or Nisga'a or Nisgaa or Nooaitch or N'quatqua or Nuxalk or Ostlq'emeylem or Pacheedath or Pauquachin or Penelakut or Qayqayt or Quatsino or Saik'uz or Samahquam or Scowlitz or Semiahmoo or Shackan or Shxwha:y or Shw'ow'hamel or Simpcw or (Siska not Karol) or Skatin or Skawahlook or Skin Tyee or Skowkale or Skuppah or Skwah or Sliammon or Soowahilie or Spallumcheen or Squiala or Stellat'en or Taku River or T'it'qet or Tla-o-qui-aht or Tlatlasikwala or Tl'azt'en or Tl'etinqox-t'in or Tlowitsis or Toosey or Toquaht or Tsartlip or Tsawataineuk or Tsawout or Tsay Keh Dene or Tseshaht or Tseycum or Tsi Del Del or Ts'kw'aylaxw or Tsleil-Waututh or T'souke or Tzeachten or Uchcklesaht or Ulkatcho or We Wai Kai or Cape Mudge or Wuikinuxv or Xaxli'p or Yaakweakwioose or Yekooche)).mp.) not (isotope* or radiocarbon* or geology* or stratigraph* or Wisconsin or Michigan or Beaver County or (Alaska not (Alaska and (Canada or British Columbia)))).mp. not (animals not (humans and animals)).sh. [mp=title, book title, abstract, original title, name of substance word, subject heading word, floating sub-heading word, keyword heading word, organism supplementary concept word, protocol supplementary concept word, rare disease supplementary concept word, unique identifier, synonyms, population supplementary concept word, anatomy supplementary concept word] | 1182 |
| 14 | (Opaskawayak or Little Saskatchewan or Fisher River Cree or Peguis or Sagkeeng or Roseau River or Norway House or Sapotaweyak or Wuskwi Siphik or Skownan or Dauphin River or Pinaymootang or Kinonjeoshtegon or O-Chi-Chak-Ko-Sipi or Tootinaowaziibeeng or Lake Manitoba or Keeseekoowenin or Waywayseecappo or Birdtail Sioux or Canupawakpa or Dakota Tipi or Brokenhead Ojibway or Northlands Nursing Station or Lac Brochet or Sayisi Cree or Tadoule Lake or Brochet or O-Pipon-Na-Piwin or South Indian Lake or Mathias Colomb or Pukatawagan or Tataskweyak or York Landing or Nisichawayasihk or Nelson House or Shamattawa or Bunibonibee or Manto Sipi or God's River or God's Lake or Red Sucker Lake or St Theresa Point or Wasagamack or Pauingassi or "Berens River" or Bloodvein).mp. or ((Sandy Bay or Long Plan or Sioux Valley or Fox Lake or War Lake or Pine Creek or Lake St Martin or Hollow Water or Little Black River or Rolling River or Dakota Plains or Swan Lake or Oxford House or Cross Lake or Split Lake or Barren Lands or Garden Hill or Poplar River or Little Grand Rapids).mp. and (exp Canada/ or Canad*.mp.)) or ((exp Indians, North American/ or exp Health Services, Indigenous/ or exp Medicine, Traditional/ or exp Shamanism/ or exp Ethnopharmacology/ or Indigenous*.mp. or Aboriginal*.mp. or Amerindian*.mp. or Autochtone*.mp. or First Nation.mp. or First Nations.mp. or Metis.mp. or (traditional adj1 (medicine* or heal* or food* or health*)).mp. or Urban Indian*.mp. or "on reserve".mp. or "off reserve*".mp. or country food*.mp. or shaman*.mp. or medicine m?n.mp. or medicine wom?n.mp. or ((native* or Indian or Indians) adj2 (person or persons or man or woman or men or women or child* or youth or youths or population* or people* or band or bands)).mp.) and (exp Manitoba/ or Manitoba*.mp. or Winnipeg.mp. or Brandon.mp. or St Boniface.mp. or Dauphin.mp. or Flin Flon.mp. or Morden.mp. or Portage la Prairie.mp. or Selkirk.mp. or Steinbach.mp. or Thompson.mp. or Winkler.mp.)) [mp=title, book title, abstract, original title, name of substance word, subject heading word, floating sub-heading word, keyword heading word, organism supplementary concept word, protocol supplementary concept word, rare disease supplementary concept word, unique identifier, synonyms, population supplementary concept word, anatomy supplementary concept word] | 703 |
| 15 | ((Ahtahkakoop or Asimakaniseekan or Amisk Lake First Nation* or Assiniboine First Nation* or Beardy or Big River First Nation* or Birch Portage or Bittern Lake or Budd's Point or Mamawetan or Carry the Kettle or Canoe Lake or Carrot River or Chicken First Nation* or Kelsey Trail Health or Sunrise Health or Prince Albert Parkland Health or Day Star or Dipper Rapids or Eagles Lake First Nation* or Elak Dase or English River First Nation* or Prairie North Health or Fishing Lake First Nation* or Flying Dust First Nation* or Fond du Lac or Four Portages or Fox Point First Nation* or Athabasca Health Authority or Gordon First Nation* or Grandmother's Bay or Hatchet Lake or Hay First Nation* or Ils a la Crosse or James Smith First Nation* or Joseph Bighead or Kawacatoose or Kahkewistahaw or Key First Nation* or Kinistin or Kinookimaw or Kitsakie or Knee Lake First Nation* or La Loche First Nation* or La Plonge or Lac La Hache or Lac La Ronge or Little Black Bear or Little Bone First Nation or Little Hills First Nation* or Little Pine First Nation* or Little Red River or Lucky Man or Makaoo or Makaw or Meadow Lake First Nation* or Ministikwan or Minoahchak or Mirond Lake or Mistawasis or Montreal Lake First Nation* or Moosomin First Nation* or Morin Lake or Mosquito-Grizzley Bear's or Muscowpetung or Muskeg Lake First Nation* or Muskeg River First Nation* or Muskoday or Muskowekwan or Nekaneet or New Thunderchild or Cypress Health or Ocean Man or Ochapowace or Okanese or Okemasis or Old Fort First Nation* or One Arrow or Onion Lake or Opawakoscikan or (Pasqua not wheat) or Peepeekisis or Pelican Lake First Nation* or Pelican Narrows First Nation* or Peter Ballantyne or Peter Pond First Nation* or Pheasant Rump or Piapot or Potato River or Poundmaker First Nation* or Primeau Lake First Nation* or Red Earth First Nation* or Red Pheasant or Sakimay or Seekaskootch or Shesheep or Standing Buffalo or Starblanket or Tumor Lake or Wahpeton or Wapachewanak or Wa-Pii or Moos-Toosis or Waterhen or Willow Bunch or Witchekan or Wood Mountain or Yellowquill or Yellow Quill or (northern Saskatchewan not (uranium or selenium))).mp. or ((exp Indians, North American/ or exp Health Services, Indigenous/ or exp Medicine, Traditional/ or exp Shamanism/ or exp Ethnopharmacology/ or (Indigenous* or Aboriginal* or Amerindian* or Autochtone* or First Nation or First Nations or Metis or Michif or Urban Indian* or "on reserve" or "off reserve*" or country food* or residential school* or shaman* or medicine m?n or medicine wom?n or Buffalo River or Island Lake or Lean Man or Pine Bluff or Salteaux or Saulteaux or Sandy Narrows or Shoal Lake or Southend or Stanley or Sturgeon or Sucker River or Sweetgrass or White Bear or White Cap or Woody Lake or Cree or Dene or Chipewyan or Dakota or Algonquian).mp. or (traditional adj1 (medicine* or heal* or food* or health*)).mp. or ((native* or Indian or Indians) adj2 (person or persons or man or woman or men or women or child* or youth or youths or population* or people* or band or bands or reserve or reserves or treaty)).mp.) and (exp Saskatchewan/ or (Saskatchewan* or Sask or Regina or Estevan or Moose Jaw or Saskatoon or Lloydminster or Swift Current).mp.))) not (exp Medicine, Chinese Traditional/ or populus.mp. or India.mp. or Wisconsin.mp. or exp Plant Extracts/ or poplar.mp. or rats.mp. or veterinary.mp. or ve.fs.) | 483 |
| 16 | ((Aakom Kiyii or Alexis Cardinal River or Alexis Elk River or Alexis Nakota Sioux or Allison Bay or Assineau River or Atikameg or Athabasca Chipweyan or Beaver First Nation or Beaver Lake Cree or Bearspaw or Beaver Ranch or Bigstone Cree or Bistcho Lake or Blood Tribe or Cadotte Lake or Carajou Settlement or Chateh or Chiniki or Chipewyan Prairie or Cold Lake First Nations or Dene Tha or Desmarais Settlement or Dog Head or Driftpile or East Prairie or Elizabeth Settlement or Enilda or Enoch Cree or Duncans First Nation or Ermineskin or Fort Chipewyan or Frog Lake or Garden Creek or Goodfish Lake or Gregoire Lake or Grouard or Heart Lake or Hobbema or Hokedhe or Horse Lake* or Janvier Health or Jere Ghalil or Jean Dor or John Dor or Kainai or Kainaa or Kapaweno or Kee Tas Kee Now or Kehewin or Ki Tue or Kapaweno or Kikino or Kinuso or Jackfish Point or Little Buffalo or Little Red River Cree or Loon Prairie or Louis Bull or Lubicon Lake Indian Nation or Maggie Willier or Makaoo or Maskwacis or Meander Health or Mikisew or Nakota or Nakoda or Namur Lake or OChiese or Old Fort or Paddle Prairie or Paul Band or Paul First Nation or Peace Point or Peavine Settlement or Peigan or Peerless Lake or Peerless Trout or Piikani or Pikuni or Pikani or Puskiakiwenin or Red Earth Creek or Saddle Lake or Samson Cree or Sarcee or Sawridge Band or Siksika or Siksikawa or Sao-kitapiiksi or Stoney First Nation or Stoney Tribe or St Isadore or Sturgeon Lake Cree or Tasttine or Tallcree or Tall Cree or Thabacha or Thebathi or Tsuu Tina or Tsu Tue or Tsu Kadhe or Unipouheos or Utikoomak or Wabasca or Wesley Band or ((Alexander or Athabasca or Beaver Lake or Blue Quill* or Cold Lake or Duncans or Enoch or Fort McKay or Fort McMurray or Loon River or Paul or Smiths Landing or Sucker Creek or Sunchild or Swan River or Whitefish Lake) adj2 (First Nation* or tribe or Indian*))).mp. or (exp Indians, North American/ or exp Health Services, Indigenous/ or exp Medicine, Traditional/ or exp Shamanism/ or exp Ethnopharmacology/ or Indigenous*.mp. or Aboriginal*.mp. or Amerindian*.mp. or Autochtone*.mp. or First Nation.mp. or First Nations.mp. or Dene.mp. or Blackfoot.mp. or Anishinaabe.mp. or Assiniboine.mp. or Metis.mp. or Mischif.mp. or Mitchif.mp. or Metif.mp. or Metchif.mp. or Bois-brule*.mp. or Mixed-blood*.mp. or Half Breed*.mp. or halfbreed*.mp. or (traditional adj1 (medicine* or heal* or food* or health*)).mp. or Urban Indian*.mp. or "on reserve".mp. or "off reserve".mp. or country food*.mp. or shaman*.mp. or medicine m?n.mp. or medicine wom?n.mp. or ((native* or Indian or Indians) adj2 (person or persons or man or woman or men or women or child* or youth or youths or population* or people* or band or bands)).mp.)) and (Beaver Lake or Brownvale or Fort McMurray or Edmonton or Calgary or Hythe or Slave Lake or Valleyview or Fort Vermilion or Morinville or Glenevis or Lac La Biche or Cold Lake or Rocky Mountain House or Duffield or Brocket or Morley or Whitecourt or Amber River or Big Horn or Buck Lake or Charles Lake or Collin Lake or Cornwall Lake or Cowper Lake or Devils Gate or Eden Valley or Fox Lake or Little Red River or Hay Lake or Lesser Slave Lake or Bonneyville or Loon Lake or Wetaskiwin or Pigeon Lake or Lake Athabasca or Fort McLeod or Barrhead or Stony Plain or Sturgeon Lake or High Prairie or Swampy Lake or Upper Hay River or Wabamun or Trout Lake or Whitefish Lake or Winefred Lake or Nordegg or Boyer River or Calling Lake or Berwyn or Fort Chipewyan or Black Diamond or Fishing Lake or Gift Lake or Kananaskis or Medicine Hat or Ponoka or Stand Off or StandOff or Alberta).mp. | 517 |
| 17 | (exp Indians, North American/ or exp Inuits/ or exp Health Services, Indigenous/ or exp Shamanism/ or exp Medicine, Traditional/ or exp Ethnopharmacology/ or American Native Continental Ancestry Group/ or (Abenaki or Abenakis or Abitibiwinni or Akwesasne* or Atikamek* or "Barriere Lake" or Betsiamite* or Cacouna or Chisasibi or Coucoucache or "Eagle Village" or Eastmain or Essipit* or Ekuanitshit* or Gespeg or Gesgapegiag* or Huron-Wendat or "Huronne Wendat" or Essipit or Inuit or Inuk or Kahnawake or Kahnawa?ke or Kanesatake or Kawawachikamach* or Kebaowek or Kipawa or Kitcisakik or Kitigan Zibi or Kuujjuaq or "Lac Romanie" or "Lac John" or "Lac Simon" or Listuguj* or "Long Point First" or Maliotenam or Maliseet or Malecite* or Mamit Innuat or Mamuitun or Manawan or Mashteuiatsh or Matimekush* or Matimekosh* or Mawiomi or Migmaw or Mig Maw or Mi?gmawei or Mingan or Mistissini* or Montagnais or Naskapi* or Natashquan* or Nemiscau or Nemaska* or Obedjiwan or Odanak or Opitciwa* or dopitciwa* or Ouje?Bougoumou or Pakuashipi* or Pessamit* or Pikogan or "Rapid Lake" or Salluit or Schefferville or Sept-Iles or Takuaikan or "Uashat Mak" or "Mani-Utenam" or Temiscaming or imiskaming or Ungava or Uashat or "Unamen Shipu" or Waban-Aki or Waskaganish or Waswanipi or Wemindji or Wemotaci or Wendat* or Wendake or Whapmagoostui or Wolinak or Kitcisakik or "Pakua Shipu" or "Pakua Shipi" or Winneway or (Peuple* adj (autochtone* or indigene* or premier* or racine* or natif*)) or "Premiere Nation" or "First Nation" or "First Nations" or Metis or Cree or Algonquin* or Algonquian* or Anishinabe* or Anishinaabeg* or autochton* or Inuit* or Innu or Innus or Innue or Micmac or Mic?Mac or Mi?gmaq or Mi?kmaq or Mowhawk or (urban adj3 (Indian* or Native* or Aboriginal*)) or (Native adj3 (american or man or men or women or woman or boy* or girl* or children or people* or indian* or Nation or tribe* or tribal or band or bands or groups or communit* or population* or health)) or indigenous* or Aboriginal* or autochtone* or treaty or on-reserve or "off reserve" or country food* or Shaman* or (traditional adj (medicine or heal*)) or residential school*).mp.) and (exp Quebec/ or (montreal or "trois rivieres" or quebec* or "james bay" or "baie james" or Laval or Gatineau or Longueuil or Sherbrooke or Saguenay or Doncaster or Levis or Terrebonne or Mascouche or l?Estrie* or Lanaudiere or l?Outaouais* or Capitale-Nationale or Chaudiere-Appalaches or Cote-Nord or Gaspe* or Mauricie or Monteregie or Laurentides or Bas-Saint-Laurent or Saint-Laurent or Nunavik).mp. or (QC or quebec).in.) | 2106 |
| 18 | ((Aamjiwnaang or Pikwakanagan or (Animbiigoo adj Zaagi?igan adj Anishinaabek*) or Wauzhushk Onigum or Naongashiing or Anishnabekwe or Anishnawbe* or "ARMSTRONG SETTLEMENT" or Aroland or ASSABASKA or "Atikameksheng Anishnawbek" or Attawapiskat or "Aundeck-Omni-Kaning" or Batchewana or "Bearfoot Onondaga" or "Biinjitiwabik Zaaging" or Bimose or "Bingwi Neyaashi" or Bkejwanong or "chapleau cree" or dokis or Eabametoong or "Fox Lake Cree" or (moravian adj2 thames) or Ginoogaming or "Kasabonika Lake" or Kashechewan or KABAPIKOTAWANGAG or Keewaytinook or Kee?Way?Win or "Kiashke Zaaging" or Kitchenuhmaykoosib or Konadaha Seneca or Koocheching or Magnetawan or Matachewan or Mattagami or "MacDowell Lake" or M?Chigeeng or Mishkeegogamang or Missanabie or Mitaanjigaming or Stanjikoming or Mocreebec or (Mohawks adj (Akwesasne or Gibson)) or "Moose Cree" or Naicatchewenin or Namaygoosisagagun or Naotkamegwanning or Neskantaga or Nibinamik or Nigigoonsiminikaaning or Nipissing or OBADJIWAN or Obashkaandagaang or "Washagamis Bay" or Ochiichagwe Bibigo?ining or Onigaming or Parmachene or Sabaskong or Sagamok or (Oneida Nation adj2 Thames) or Shawanaga or Sheguiandah or Sheshegwaning or Taykwa Tagamou or Temagami or Wabaseemoong or Wabauskang or "Wabigoon Lake" or Wahgoshig or Wahnapitae or "Wahta Mohawks" or Wapekeka or Wasauksing or Wauzhushk Onigum or Wawakepewin or Webequie or Weenusk or Wikwemikong or ((Ardoch or Algonquin or Beausoleil or "big grassy" or "Iskatewizaagegan 39 Independent" or beaverhouse or "brunswick house" or "buffalo point" or caldwell or "cat lake" or Couchiching or "Curve Lake" or "deer lake" or delaware or "duck lake" or "Eagle Lake" or Thames or "Fort William" or "Garden River" or "Grassy Narrows" or Hiawatha or Henvey Inlet or Hornepayne or "Gull Bay" or "King Fisher" or (Lac adj Mille adj Lacs) or (Lac adj Croix) or "Long Lake" or Magnetawan or "Marten Falls" or "Martin Falls" or Mississauga or "New Credit" or "Moose Deer Point" or Munsee?Delaware or "Muskrat Dam" or "North Caribou Lake" or "North Spirit Lake" or Northwest Angle or Sioux Narrows or Pays Plat or Pic Mobert or "Red Rock" or "Sachigo Lake" or "Sandy Lake" or "Savant Lake" or Saugeen or Seine River or Serpent River or "Shoal Lake" or Stony Point or Stoney Point or "Grand River Territory" or "Slate Falls" or Whitefish River or Whitesand or "Whitewater Lake" or "Wunnumin Lake") adj2 first nation*)).mp. or (exp Indians, North American/ or exp Inuits/ or exp Health Services, Indigenous/ or exp Shamanism/ or exp Medicine, Traditional/ or exp Ethnopharmacology/ or American Native Continental Ancestry Group/ or (Peuple adj (autochtones or indidgenes or premier or racing or natif*)).mp. or ("Premiere Nation" or "First Nation" or "First Nations" or Metis or chippewas or Cree or Algonquin* or Algonquian* or Anishinabe* or Anishnabeg or Anishinaabe* or autochthon* or Inuit* or Innu or Innus or Innue or Micmac or Mic?Mac or Mi?gmaq or Mi?kmaq or Mowhawk or Ojibw* or Cayuga).mp. or (urban adj3 (Indian* or Native* or Aboriginal*)).mp. or ((Native not (bacteri* or plant* or species or micro* or biot* or strain or strains or probiotic or zoo* or geno*)) adj3 (american or man or men or women or woman or boy* or girl* or children or people* or indian* or Nation or tribe* or tribal or band or bands or groups or communit* or population* or health)).mp. or ((indigenous* not (bacteri* or plant* or species or micro* or biot* or strain or strains or probiotic or zoo* or geno*)) or Aboriginal* or (autochon* not (bacteri* or plant* or species or micro* or biot* or strain or strains or probiotic or zoo* or geno*)) or treaty or on-reserve or off-reserve or country food* or Shaman* or (traditional adj (medicine or heal*)) or residential school*).mp.)) and (exp Ontario/ or (ontario or toronto or ottawa or sarnia or london or hamilton or windsor or roseneath or "golden lake" or beardmore or "christian island" or "Cedar Point" or morson or "bay of quinte" or "bear island" or "Bearskin Lake" or "whitefish lake" or naughton or "little current" or "Kirkland lake" or "big island" or macdiarmid or "thunder bay" or wallaceburg or chapleau or leamington or "georgina island" or "CAPE CROKER" or "FOX LAKE" or "CHIEF’S POINT" or "Constance Lake" or "Curve Lake" or "deer lake" or "duck lake" or "Eagle Lake" or "ENGLISH RIVER" or Rama or Erie St?Clair or "FACTORY ISLAND" or "Flying Post" or "FORT ALBANY" or "FORT HOPE" or "Fort Severn" or "Fort William" or Wiarton or Southampton or Saugeen or Muncy or Calstock or "Fort Frances" or "FRENCH RIVER" or Thamesville or Monteville or "Eabamet Lake" or Nipigon or "Georgian Bay" or "Long Lac" or "Long Lake" or Grassy Narrows or Keene or Pickerel or Hornepayne or Glebe Farm or "GOULAIS BAY" or Grey? Owen Sound or GROS CAP or "Shoal Lake" or Kashechewan or INDIAN RIVER or "King Fisher Lake" or "Big Trout Lake" or "Sandy Lake" or "Fort Frances" or "Lac Seul" or "LAKE HELEN" or (LAKE adj WOODS) or Hudson or Britt or Okoki Post or "MANITOU RAPIDS" or gogama or "McDowell Lake" or "Red Lake" or Wawa or "New Osnaburgh" or "Garden River" or "Blind River" or Hagersville or "Port Perry" or "Moose Factory" or Cornwall or Deseronto or Mactier or Muncey or Delvin or Pawitik or "Whitefish Bay" or "Landsdowne House" or "Summer Beaver" or "Weagamow Lake" or "North Spirit Lake" or "Rainy Lake" or "Rainy River" or "Sioux Narrows" or Keewating or Kenora or "Sault Ste Marie" or "Nestor Falls" or "Heron Bay" or Southwold or "Pays Plat" or Mobert or "Pic River" or "Moon River" or Massey or "Sachigo Lake" or "Savant Lake" or Cutler or Nobel or Sheshegwaning or Kejick or Ohsweken or "Slate Falls" or Cochrane or "Lake Tamagami" or Thessalon or Whitedog or "Ear Falls" or Dryden or Matheson or Bala or Capreol or "Angling Lake" or "Parry Sound" or "Sioux Lookout" or Webequie or Peawanuk or "Birch Island" or Armstrong or "Wunnumin Lake" or Silverwater or Walpole Island or WINISK).mp.) | 2794 |
| 19 | ((Carcross or (Tagish not meteorite*) or Champagne First Nation or Aishihik or Ehdiitat or Nacho Nyak Dun or Gwichya or Little Salmon or Carmacks or Nihtat or Selkirk First Nation or Ta'an Kwach'an or Tetlitn or Tr'ondek Hwech'in or White River First Nation or Vuntut or Yellowknives or (Hare adj2 (man or men or woman or women or child* or youth* or adult* or people* or person or persons or tribe or tribal or band or bands)) or Tanana or Tanana or Tutchone* or Denesuline or Tahltan or MacKenzie Valley or Old Crow or "Upper Liard" or "Eagle Plains" or "Keno City" or Carcross or Teslin or "Fort Selkirk" or Carmacks or Haines Junction or Dawson City).mp. or ((Canad*.mp. or exp Canada/) and (Beaver Creek or Pelly or Destruction Bay or Watson Lake).mp.) or ((exp Indians, North American/ or exp Health Services, Indigenous/ or exp Medicine, Traditional/ or exp Shamanism/ or exp Ethnopharmacology/ or Indigenous*.mp. or Aboriginal*.mp. or Amerindian*.mp. or Autochtone*.mp. or Metis.mp. or First Nation.mp. or First Nations.mp. or exp Inuit/ or Inuit*.mp. or Chipewyan.mp. or Kaska.mp. or Kaskas.mp. or Tlingit.mp. or Dene.mp. or Gwich'in.mp. or Gwichin.mp. or Gwitchin.mp. or Kutchin*.mp. or Sahtu.mp. or Tlicho.mp. or Tli Cho.mp. or (traditional adj1 (medicine* or heal* or food* or health*)).mp. or Urban Indian*.mp. or "on reserve".mp. or "off reserve*or country food*".mp. or shaman*.mp. or medicine m?n.mp. or medicine wom?n.mp. or treaty.mp. or treaties.mp. or ((native* or Indian or Indians) adj2 (person or persons or man or woman or men or women or child* or youth or youths or population* or people* or band or bands)).mp.) and (exp Yukon Territory/ or Yukon*.mp. or ((Beaufort Sea or Whitehorse) and Canad*).mp.))) not ((exp Alaska/ or Alaska*.mp.) not ((exp Alaska/ or Alaska*.mp.) and (exp Yukon/ or Yukon*.mp.))) not (Yukon-Kuskok* or lepus or geology* or stratigraphi* or subduction* or volcan* or Holocene or pleistocene).mp. [mp=title, book title, abstract, original title, name of substance word, subject heading word, floating sub-heading word, keyword heading word, organism supplementary concept word, protocol supplementary concept word, rare disease supplementary concept word, unique identifier, synonyms, population supplementary concept word, anatomy supplementary concept word] | 163 |
| 20 | ((((exp Medicine, Traditional/ not Chinese.mp.) or exp Shamanism/ or exp Indians, North American/ or exp Inuits/ or exp Health Services, Indigenous/ or exp Ethnopharmacology/ or (Inuit* or Eskimo* or Esquimau* or Athapaskan or Gwich'in or Metis or Inuvialuktun or Cree or Aboriginal* or Indigenous* or off-reserve or on-reserve or First Nation or First Nations or Amerindian or (urban adj3 (Indian* or Native* or Aboriginal*)) or ethnomedicine or country food* or residential school* or shaman* or traditional medicine* or traditional heal* or traditional food* or medicine man or medicine woman or autochtone* or treaty or (Native adj1 (man or men or women or woman or boy* or girl* or adolescent* or youth or youths or person* or adult or people* or Indian* or Nation or tribe* or tribal or band or bands))).mp.) and (exp Northwest Territories/ or Northwest Territories.mp. or NWT.mp. or Yellowknife.mp. or Western Arctic.mp.)) or (Aklavik or Banks Island or Behchoko or Rae Edzo or Colville Lake or De Cho or Deline or Denendeh or Fort Good Hope or Fort Liard or Fort McPherson or Fort McPherson or Fort Providence or Fort Providence or Fort Simpson or Fort Smith or Gameti or Hay River or Inuvik or Jean Marie River or Lutselk'e or Norman Wells or Paulatuk or Sachs Harbour or Trout Lake or Tsiigehtchic or Tuktoyaktuk or Tulita or Tulit'a or Ulukhaktok or Victoria Island or Whati or Wha Ti or Wrigley or (Hare adj2 (man or men or boy or boys or girl or girls or adult* or youth* or adolescent* or Nation or people* or Indians* or tribe* or tribal*)) or Slavey or Chipewyan or Tlicho or Dogrib or Yellowknives or Dene or Sahtu or Inuvaluit* or Inuinnaqtun).mp.) not ((fort smith adj1 ar*).mp. or ((rabbit* or lepus or lemming* or fox or foxes or wolf or wolves or (wrigley adj1 (n or g or forcep*))).mp. or ve.fs.)) [mp=title, book title, abstract, original title, name of substance word, subject heading word, floating sub-heading word, keyword heading word, organism supplementary concept word, protocol supplementary concept word, rare disease supplementary concept word, unique identifier, synonyms, population supplementary concept word, anatomy supplementary concept word] | 699 |
| 21 | (exp Nunavut/ or Nunavut.mp. or Eastern Arctic.mp. or Alert Bay.mp. or Alexandra Fiord.mp. or Amadjuak.mp. or Aquiatulavik Point.mp. or Arctic Bay.mp. or Arviat.mp. or Baffin Island.mp. or Baker Lake.mp. or Bathurst Inlet.mp. or Belcher Islands.mp. or Bylot Island.mp. or Cambridge Bay.mp. or Iqaluktuttiaq.mp. or Cape Dorset.mp. or Cape Dyer.mp. or Cape Smith.mp. or Charlton Depot.mp. or Chesterfield Inlet.mp. or Clyde River.mp. or Coral Harbour.mp. or Craig Harbour.mp. or Dundas Harbor.mp. or Ellesmere Island.mp. or Ennadai.mp. or Eskimo Point.mp. or Fort Conger.mp. or Fort Hope.mp. or Fort Ross.mp. or Gjoa Haven.mp. or Grise Fiord.mp. or Hall Beach.mp. or Hazen Camp.mp. or Igloolik.mp. or Ikaluit.mp. or Iqaluit.mp. or Isachsen.mp. or Kekerten.mp. or Kimmirut.mp. or King William Island.mp. or Kipisa.mp. or Kitikmeot o r Kivalliq.mp. or Kivitoo.mp. or Kugaaruk.mp. or Kugluktuk.mp. or Maguse River.mp. or Nanasivik.mp. or Nottingham Island.mp. or Nuwata.mp. or Padlei.mp. or Padloping Island.mp. or Pangnirtung.mp. or Perry Island.mp. or Pond Inlet.mp. or Port Burwell.mp. or Qoloqtaaluk.mp. or Qikiqtarjuaq.mp. or Rankin Inlet.mp. or Read Island.mp. or Repuilse Bay.mp. or Resolute Bay.mp. or Resolution Island.mp. or Sanikiluak.mp. or Taloyoak.mp. or Tanquary Camp.mp. or Tavani.mp. or Thom Bay.mp. or Umingmaktok.mp. or Victoria Island.mp. or Wager Bay.mp. or Whale Cove.mp. or Eastern Arctic.mp. or ((Lupin or Polaris or Eureka or Fullerton) and Canad*).mp.) not (exp behavior, animal/ or exp ecosystems/ or exp endangered species/ or (sediment* or mantle or basalt* or cretaceous* or fossil* or paleo* or geolog* or stratigraph* or glaci* or refugia* or moraine* or pliocene or gravity or methylmercury or hydrolog* or hydrogeol* or volcan* or mesospher* or inferomet* or habitat* or animal behavior* or endangered species).mp.) [mp=title, book title, abstract, original title, name of substance word, subject heading word, floating sub-heading word, keyword heading word, organism supplementary concept word, protocol supplementary concept word, rare disease supplementary concept word, unique identifier, synonyms, population supplementary concept word, anatomy supplementary concept word] | 819 |
| 22 | (exp Indians, North American/ or exp Health Services, Indigenous/ or Metis.mp. or exp Medicine, Traditional/ or exp Shamanism/ or exp Ethnopharmacology/ or Indigenous*.mp. or Aboriginal*.mp. or Amerindian*.mp. or Autochtone*.mp. or Metis.mp. or First Nations.mp. or First Nation*.mp. or (traditional adj1 (medicine* or heal* or food* or health*)).mp. or Urban Indian*.mp. or "on reserve".mp. or "off reserve*".mp. or country food*.mp. or residential school*.mp. or shaman*.mp. or medicine m?n.mp. or medicine wom?n.mp. or ((native* or Indian or Indians) adj2 (person or persons or man or woman or men or women or child* or youth or youths or population* or people* or band or bands)).mp. or Montagnais.mp. or Maliseet.mp. or Naskapi*.mp. or Mi'kmaq.mp. or Micmac.mp. or Mic mac.mp. or Migmaw.mp. or Mig maw.mp. or Beothuk*.mp.) and (((Fredrickton or Moncton or New Jersey).mp. and (exp Canada/ or Canad*.mp.)) or exp New Brunswick/ or (New Brunswick* not ("New Brunswick NJ" or New Jersey or ferment*)).mp. or Nouveau Brunswick.mp. or Big Hole Tract.mp. or Metepenagiag.mp. or Eel Ground First Nation.mp. or Buctouche.mp. or Esgenoopetitj.mp. or Burnt Church.mp. or Devon Reserve.mp. or St Mary's First Nation.mp. or Eel River Reserve.mp. or Eel River Bar.mp. or Fort Folly Indian Point Reserve.mp. or Indian Island First Nation.mp. or Indian Ranch Reserve.mp. or Kingsclear.mp. or St John River Valley Tribal Council.mp. or Oromocto.mp. or Pabineau.mp. or Pokemouche.mp. or Mawiw.mp. or (Red Bank adj2 Reserve).mp. or Richibucto.mp. or St Basile.mp. or Madawaska.mp. or Soegao.mp. or Tabusintac.mp. or Tobique.mp. or Wolastoqiyik.mp. or Woodstock First Nation.mp.) [mp=title, book title, abstract, original title, name of substance word, subject heading word, floating sub-heading word, keyword heading word, organism supplementary concept word, protocol supplementary concept word, rare disease supplementary concept word, unique identifier, synonyms, population supplementary concept word, anatomy supplementary concept word] | 25 |
| 23 | (((((Indigenous* or Aboriginal* or Amerindian* or Autochtone* or First Nation or First Nations or (traditional adj1 (medicine* or heal* or food* or health*)) or Urban Indian* or "on reserve" or "off reserve*" or country food* or shaman* or medicine m?n or medicine wom?n or (native* or Indian or Indians)) adj2 (person or persons or man or woman or men or women or child* or youth or youths or population* or people* or band or bands)) or Montagnais or Maliseet or Naskapi* or Mi'kmaq or Micmac or Mic mac or Migmaw or Mig maw or Beothuk*).mp. or exp Health Services, Indigenous/ or exp Indians, North American/ or Metis.mp. or exp Medicine, Traditional/ or exp Shamanism/ or exp Ethnopharmacology/) and (((Wolfville or Middleton or Kentville or Berwick or Inverness or New Waterford or Sackville or Springhill or Halifax or Dartmouth or Truro or New Glasgow or Sydney or Canso or Guysborough or Parrsboro or Pictou or Liverpool or Lunenburg or Amherst) and Canad*).mp. or exp Nova Scotia/ or Nova Scotia*.mp. or Nouvelle Ecosse.mp. or Pictou Landing.mp. or Bear River.mp. or Boat Harbour.mp. or Annapolis Royal.mp. or Antigonish.mp. or Baddeck.mp. or Cheticamp.mp. or Cape Breton.mp. or Neil's Harbour.mp. or Glace Bay.mp. or Tatamagouche.mp. or Sheet Harbour.mp. or Cambridge Reserve.mp. or Annapolis Valley First Nation.mp. or Chapel Island First Nation.mp. or Cole Harbour.mp. or Eskasoni.mp. or Fisher's Grant.mp. or Franklin Manor.mp. or Paq'tnkek.mp. or (Glooscap adj1 (First Nation or reserve)).mp. or Acadia First Nation.mp. or Gold River Reserve.mp. or Horton Reserve.mp. or Shubenacadie First Nation.mp. or Indian Brook Reserve.mp. or Wagmatcook.mp. or Waycobah.mp. or Millbrook First Nation.mp. or Malagawatch.mp. or Medway River.mp. or Membertou.mp. or Merigomish.mp. or Musquodoboit.mp. or New Ross Reserve.mp. or Pennal Reserve.mp. or Pomquet.mp. or Poonhook.mp. or Sheet Harbour.mp. or St Croix Reserve.mp. or Summerside Reserve.mp. or Sydney Reserve.mp. or Truro Reserve.mp. or We'koqma'q.mp. or Wycocomagh.mp. or Wildcat Reserve.mp. or Yarmouth Reserve.mp.)) not (geology or geologic or stratigraphy* or animal* or cat or cats or kitten or deer or bird* or dog or dogs or feline or canine or bovine or equine or porcine or pig or piglet or swine or rat or rats or horse or horses or mouse or mice).mp. [mp=title, book title, abstract, original title, name of substance word, subject heading word, floating sub-heading word, keyword heading word, organism supplementary concept word, protocol supplementary concept word, rare disease supplementary concept word, unique identifier, synonyms, population supplementary concept word, anatomy supplementary concept word] | 49 |
| 24 | ((exp Indians, North American/ and Canad*.mp.) or Indigenous Canadians/ or exp Inuits/ or exp Health Services, Indigenous/ or exp Ethnopharmacology/ or (Athapaskan or Saulteaux or Wakashan or Cree or Dene or Inuit or Inuk or Inuvialuit* or Haida or Ktunaxa or Tsimshian or Gitxsan or Gitksan or "Nisga'a" or Haisla or Heiltsuk or Oweenkeno or "Kwakwaka'wakw" or "Nuu chah nulth" or "Tsilhqot'in" or Dakelh or "Wet'suwet'en" or Sekani or Dunne-za or Dene or Tahltan or Kaska or Tagish or Tutchone or Nuxalk or Salish or St'at'imc or Stl'atl'imx or Stl'atl'imc or Nlaka'pamux or Okanagan or "Sec wepmc" or Secwepemc or Tlingit or Anishinaabe or Blackfoot or Nakoda or Tasttine or "Tsuu T'ina" or "Tsuut'ina" or "Gwich'in" or Han or Algonquin or Nipissing or Ojibwa or Potawatomi or Innu or Maliseet or "Mi'kmaq" or Micmac or Passamaquoddy or Haudenosaunee or Cayuga or Mohawk or Oneida or Onondaga or Seneca or Tuscarora or Wyandot or Aboriginal* or Indigenous* or Metis or red road or "on reserve" or off-reserve or First Nation or First Nations or Amerindian).mp. or (urban adj3 (Indian* or Native* or Aboriginal*)).mp. or ethnomedicine.mp. or country food*.mp. or residential school*.mp. or ((exp Medicine, Traditional/ or traditional medicine*.mp.) not Chinese.mp.) or exp Shamanism/ or shaman*.mp. or traditional heal*.mp. or traditional food*.mp. or medicine man.mp. or medicine woman.mp. or autochtone*.mp. or (Native* adj1 (man or men or women or woman or boy* or girl* or adolescent* or youth or youths or person* or adult or people* or Indian* or Nation or tribe* or tribal or band or bands)).mp.) and (exp Canada/ or (Canad* or British Columbia or Colombie Britannique or Alberta or Saskatchewan or Manitoba or Ontario or Quebec or Nova Scotia or New Brunswick or Newfoundland or Labrador or Prince Edward Island or Yukon Territory or NWT or Northwest Territories or Nunavut or Nunavik or Nunatsiavut or NunatuKavut).mp.) [mp=title, book title, abstract, original title, name of substance word, subject heading word, floating sub-heading word, keyword heading word, organism supplementary concept word, protocol supplementary concept word, rare disease supplementary concept word, unique identifier, synonyms, population supplementary concept word, anatomy supplementary concept word] | 8958 |
| 25 | 12 or 13 or 14 or 15 or 16 or 17 or 18 or 19 or 20 or 21 or 22 or 23 or 24 | 12817 |
| 26 | Disability Evaluation/ | 51617 |
| 27 | Disabled Persons/ | 48397 |
| 28 | "Activities of Daily Living"/ | 74146 |
| 29 | "Quality of Life"/ | 281757 |
| 30 | Functional Status/ | 1370 |
| 31 | Mobility Limitation/ | 5307 |
| 32 | Physical Functional Performance/ | 2818 |
| 33 | exp "International Classification of Functioning, Disability and Health"/ | 880 |
| 34 | (disabilit* or disabl*).ti,ab,kw. | 296075 |
| 35 | impair*.ti,ab,kw. | 884283 |
| 36 | (functioning* or functional*).ti,ab,kw. | 2050231 |
| 37 | (activit* adj2 (limit* or restrict*)).ti,ab,kw. | 19952 |
| 38 | (participat* adj2 (limit* or retrict*)).ti,ab,kw. | 1969 |
| 39 | (function* adj2 (outcome* or limit* or restrict*)).ti,ab,kw. | 91262 |
| 40 | (mobility* adj3 (restrict* or limit* or loss* or lose* or losing* or lost*)).ti,ab,kw. | 9437 |
| 41 | (restrict* adj2 back).ti,ab,kw. | 60 |
| 42 | (HRQOL* or HAQ or Health Assessment Questionnaire).ti,ab,kw. | 29902 |
| 43 | (PROMIS or Patient Reported Outcomes Measurement Information System).ti,ab,kw. | 4699 |
| 44 | (PROM or PROMS or patient reported outcome measure).ti,ab,kw. | 10558 |
| 45 | (PSFS or Patient Specific Functional Scale).ti,ab,kw. | 986 |
| 46 | oswestry*.ti,ab,kw. | 10311 |
| 47 | Roland-Morris*.ti,ab,kw. | 1762 |
| 48 | (WHO-DAS* or WHODAS* or WHO DAS).ti,ab,kw. | 1016 |
| 49 | Pain Disability Index.ti,ab,kw. | 528 |
| 50 | 26 or 27 or 28 or 29 or 30 or 31 or 32 or 33 or 34 or 35 or 36 or 37 or 38 or 39 or 40 or 41 or 42 or 43 or 44 or 45 or 46 or 47 or 48 or 49 | 3294843 |
| 51 | Chronic Pain/ | 23812 |
| 52 | exp Pain/ | 470460 |
| 53 | exp Neuralgia/ | 25328 |
| 54 | Sciatica/ | 5219 |
| 55 | Pain Management/ | 41733 |
| 56 | Pain Clinics/ | 1635 |
| 57 | Pain Measurement/ | 95085 |
| 58 | (pain* or allodyn* or analges* or anesth* or anaesth* or causalg* or dysesthes* or hyperalg* or hyperesthes* or hyperpath* or hypoalges* or hypoesthes* or neuralg* or ache or aches or headache* metatarsalg* or arthralg* or neuritis* or neuropath* or nocicept* or "noxious stimulus" or paresthes* or sensitization* or emotion* or mental* or psycholog* or physical* or intergenerat* or spirit*).ti,ab,kw. | 3236355 |
| 59 | 51 or 52 or 53 or 54 or 55 or 56 or 57 or 58 | 3376731 |
| 60 | 25 and (50 or 59) | 2736 |
| 61 | (comment or editorial or letter or review or systematic review or guideline or practice guideline or case reports or randomized controlled trial).pt. | 8222872 |
| 62 | exp Animals/ | 26955814 |
| 63 | exp Humans/ | 21761956 |
| 64 | 62 not 63 | 5193858 |
| 65 | 60 not (61 or 64) | 2008 |

**Appendix B: Strategies for searching grey literature**

| **Strategy 1: targeted website searching** | *Key websites pertaining to Indigenous organizations* | 14 websites  Including the National Collaborating Centre for Indigenous Health, First Nations, Inuit, and Métis specific organizational websites, and governmental health-related websites from CADTH Grey Matters Checklist |
| --- | --- | --- |
| **Strategy 2: grey literature database search** | *Key grey literature database and collections which publish Indigenous-specific content* | 3 databases  ProQuest Dissertations and Theses Global database  ArcticNet Publications  Indigenous Studies Portal |
| **Strategy 3: search engine searching** | *Relevant search engines searched using a combination of different search terms (in each search, first 100 items screened for relevance to research question)* | Google Scholar  Google search engine |
| **Strategy 4: knowledge experts** | *Recommendation of key websites and databases by content experts* | 5 websites  Additional national, Indigenous and pain-specific websites recommended by members of the IAC |

**Appendix C: Studies ineligible following full-text review**

1. Abbey, S. E., Hood, E., Young, L. T., & Malcolmson, S. (1991). New perspectives on mental health problems in Inuit women. *Arctic Medical Research*, 285-287.

*Reason for exclusion:* Not reporting pain-related disability

1. Abbey, S. E., Hood, E., Young, L. T., & Malcolmson, S. A. (1993). Psychiatric consultation in the Eastern Canadian Arctic: III. Mental health issues in Inuit women in the Eastern Arctic. *The Canadian Journal of Psychiatry*, *38*(1), 32-35.

*Reason for exclusion:* Not reporting pain-related disability

1. Aboyeji, A. (2021). *The Association Between Ethnicity and Caregiver Health* (Master's thesis, The University of Western Ontario (Canada)).

*Reason for exclusion:* Not reporting pain-related disability

1. Adelson, N. (2000). Re-imagining Aboriginality: An Indigenous peoples’ response to social suffering. *Transcultural psychiatry*, *37*(1), 11-34. <https://dx.doi.org/10.1177/136346150003700101>

*Reason for exclusion:* Not reporting pain-related disability

1. Affleck, W., Oliffe, J. L., Inukpuk, M. M., Tempier, R., Darroch, F., Crawford, A., & Séguin, M. (2022). Suicide amongst young Inuit males: The perspectives of Inuit health and wellness workers in Nunavik. *SSM-Qualitative Research in Health*, *2*, 100069.
   <https://dx.doi.org/10.1016/j.ssmqr.2022.100069>

*Reason for exclusion:* Not reporting pain-related disability

1. Ahmad, A., & Gosling, J. (2021). The remoteness of pain in Canada's Indigenous peoples' collective memory. *The Lancet*, *398*(10303), 841-842. DOI: 10.1016/S0140-6736(21)01685-8

*Reason for exclusion:* Not an epidemiological study, qualitative or mixed-methods

1. Ahmed, A., Hakim, A., & Becker, A. (2018). Evaluation of eczema, asthma, allergic rhinitis and allergies among the grade-1 children of Iqaluit. *Allergy, Asthma & Clinical Immunology*, *14*, 1-14.
   <https://dx.doi.org/10.1186/s13223-018-0232-2>

*Reason for exclusion:* Not reporting pain-related disability

1. Barkwell, D. (2000). *On Ojibway cultural perspective on the illness cancer and related pain*. (Doctoral thesis, University of Manitoba (Canada)).

*Reason for exclusion:* Not reporting pain-related disability

1. Barkwell, D. (2005). Cancer pain: Voices of the Ojibway people. *Journal of Pain and Symptom Management*, *30*(5), 454-464.

*Reason for exclusion:* Not reporting pain-related disability

1. Barnabe, C., Elias, B., Bartlett, J., Roos, L., & Peschken, C. (2008). Arthritis in Aboriginal Manitobans: evidence for a high burden of disease. *The Journal of rheumatology*, *35*(6), 1145-1150.

*Reason for exclusion:* Not reporting pain-related disability

1. Barnabe, C., Hemmelgarn, B., Jones, C. A., Peschken, C. A., Voaklander, D., Joseph, L., ... & Marshall, D. A. (2015). Imbalance of prevalence and specialty care for osteoarthritis for first nations people in Alberta, Canada. *The Journal of rheumatology*, *42*(2), 323-328.

*Reason for exclusion:* Not reporting pain-related disability

1. Barnabe, C., Crane, L., White, T., Hemmelgarn, B., Kaplan, G. G., Martin, L., & Maksymowych, W. P. (2018). Patient-reported outcomes, resource use, and social participation of patients with rheumatoid arthritis treated with biologics in Alberta: experience of Indigenous and non-Indigenous patients. *The Journal of Rheumatology*, *45*(6), 760-765.

*Reason for exclusion:* Not reporting pain-related disability

1. Baron, M., Riva, M., Fletcher, C., Lynch, M., Lyonnais, M. C., & Laouan Sidi, E. A. (2021). Conceptualisation and operationalisation of a holistic indicator of health for older Inuit: results of a sequential mixed-methods project. *Social Indicators Research*, *155*, 47-72. <https://dx.doi.org/10.1007/s11205-020-02592-5>

*Reason for exclusion:* Not reporting pain-related disability

1. Bath, B., Trask, C., McCrosky, J., & Lawson, J. (2014). A Biopsychosocial Profile of Adult Canadians with and without Chronic Back Disorders: A Population‐Based Analysis of the 2009‐2010 Canadian Community Health Surveys. *BioMed research international*, *2014*(1), 919621.
   <https://dx.doi.org/10.1155/2014/919621>

*Reason for exclusion:* Not reporting pain-related disability

1. Beaudin, P. G. (2012). *A contemporary socio-cultural exploration of health and healing: perspectives from members of the Oneida Nation of the Thames*. (Doctoral thesis, The University of Western Ontario (Canada)).

*Reason for exclusion:* Not reporting pain-related disability

1. Benoit, A. C., Cotnam, J., Raboud, J., Greene, S., Beaver, K., Zoccole, A., ... & Loutfy, M. (2016). Experiences of chronic stress and mental health concerns among urban Indigenous women. *Archives of women's mental health*, *19*, 809-823. DOI: 10.1007/s00737-016-0622-8

*Reason for exclusion:* Not reporting pain-related disability

1. Boag, T. J. (1970). Mental health of native peoples of the Arctic. *Can Psychiatr Assoc J, 15*(2), 115-120. DOI: 10.1177/070674377001500203

*Reason for exclusion:* Not an epidemiological study, qualitative or mixed-methods

1. Bougie, E. (2009). *Aboriginal Peoples Survey, 2006: School experiences of off-reserve First Nations children aged 6 to 14*. Statistics Canada, Social and Aboriginal Statistics Division.

*Reason for exclusion:* Not reporting pain-related disability

1. Bourassa, C., Blind, M., Dietrich, D., & Oleson, E. (2015). Understanding the intergenerational effects of colonization: Aboriginal women with neurological conditions—their reality and resilience. *International Journal of Indigenous Health*, *10*(2), 3-20.

*Reason for exclusion:* Not reporting pain-related disability

1. Bowd, A. D. (2005). Otitis media: health and social consequences for aboriginal youth in Canada’s north. *International Journal of Circumpolar Health*, *64*(1), 5-15. DOI:
   10.3402/ijch.v64i1.17949

*Reason for exclusion:* Not an epidemiological study, qualitative or mixed-methods

1. Bruce, S.G. (1999). *Prevalence, risk factors and impact of diabetes among the western Canadian Métis*. (Doctoral dissertation, University of Manitoba).

*Reason for exclusion:* Not reporting pain-related disability

1. Bruce, S. G. (2000). The impact of diabetes mellitus among the Métis of western Canada. *Ethnicity & Health*, *5*(1), 47-57.

*Reason for exclusion:* Not reporting pain-related disability

1. Bruce, S.G., Kliewer, E.V., Young, T.K., Mayer, T., Wajda, A. (2003). Diabetes among the Métis of Canada: Defining the population, estimating the disease. *Canadian Journal of Diabetes*, *27*(4), 442-448.

*Reason for exclusion:* Not reporting pain-related disability

1. Cameron, C. M., Purdie, D. M., Kliewer, E. V., & McClure, R. J. (2005). Differences in prevalence of pre-existing morbidity between injured and non-injured populations. *Bulletin of the World Health Organization*, *83*(5), 345-352.

*Reason for exclusion:* Non-Indigenous population and not in Canada

1. Cañizares, M., Power, J. D., Perruccio, A. V., & Badley, E. M. (2008). Association of regional racial/cultural context and socioeconomic status with arthritis in the population: a multilevel analysis. *Arthritis Care & Research*, *59*(3), 399-407. <https://dx.doi.org/10.1002/art.23316>

*Reason for exclusion:* Not reporting pain-related disability

1. Caron, J., & Liu, A. (2010). A descriptive study of the prevalence of psychological distress and mental disorders in the Canadian population: comparison between low-income and non-low-income populations. *Health Promotion and Chronic Disease Prevention in Canada*, *30*(3).

*Reason for exclusion:* Not reporting pain-related disability

1. Chahar Mahali, S., Beshai, S., & Wolfe, W. L. (2021). The associations of dispositional mindfulness, self-compassion, and reappraisal with symptoms of depression and anxiety among a sample of Indigenous students in Canada. *Journal of American College Health*, *69*(8), 872-880.
   <https://dx.doi.org/10.1080/07448481.2020.1711764>

*Reason for exclusion:* Not reporting pain-related disability

1. Chai, L. (2024). Disability and suicidal ideation among Indigenous adults in Canada: cultural resources as contingencies. *Archives of suicide research*, *28*(2), 610-628. <https://dx.doi.org/10.1080/13811118.2023.2199803>

*Reason for exclusion:* Not reporting pain-related disability

1. Chen, A., Dyck Holzinger, S., Oskoui, M., Shevell, M., Canadian Cerebral Palsy Registry, Andersen, J. (2021). Cerebral palsy in Canadian Indigenous children. *Developmental Medicine & Child Neurology*, *63*(5), 614-622. <https://dx.doi.org/10.1111/dmcn.14776>

*Reason for exclusion:* Not reporting pain-related disability

1. Clouston, J. E. (2007). *A qualitative study of experiences of Aboriginal caregivers of children with developmental disabilities*. (Doctoral thesis, Wilfrid Laurier University (Canada)).

*Reason for exclusion:* Not reporting pain-related disability

1. Crowshoe, L., Dannenbaum, D., Green, M., Henderson, R., Hayward, M. N., Toth, E., & Diabetes Canada Clinical Practice Guidelines Expert Committee. (2018). Type 2 diabetes and Indigenous peoples. *Canadian journal of diabetes*, *42*, S296-S306. DOI:
   10.1016/j.jcjd.2017.10.022

*Reason for exclusion:* Not an epidemiological study, qualitative or mixed-methods

1. Deodhar, J. K., Noronha, V., Muckaden, M. A., Atreya, S., Joshi, A., Tandon, S. P., ... & Prabhash, K. (2017). A study to assess the feasibility of introducing early palliative care in ambulatory patients with advanced lung cancer. *Indian Journal of Palliative Care*, *23*(3), 261. DOI: 10.4103/IJPC.IJPC_19_17

*Reason for exclusion:* Non-Indigenous population and not in Canada

1. Destounis, B. V., MacDougall, J. C., Gisel, E., Pollitt, T., Watters, G., & Gledhill, R. (1991). The prevalence of disability in the Baffin: a model for the delivery of community-based rehabilitation. *Arctic medical research*, 30-33.

*Reason for exclusion:* Not reporting pain-related disability

1. Dosman, J. A., Karunanayake, C. P., Fenton, M., Ramsden, V. R., Skomro, R., Kirychuk, S., ... & Pahwa, P. (2021). Prevalence of insomnia in two Saskatchewan First Nation communities. *Clocks & Sleep*, *3*(1), 98-114. DOI: <https://dx.doi.org/10.3390/clockssleep3010007>

*Reason for exclusion:* Not reporting pain-related disability

1. Durst, D., Bluechardt, M. H. S., Morin, G., & Rezansoff, M. (2001). *Urban aboriginal persons with disabilities: Triple Jeopardy!*. Regina: Social Policy Research Unit, University of Regina.

*Reason for exclusion:* Not reporting pain-related disability

1. Durst, D., South, S. M., & Bluechardt, M. (2006). Urban First Nations people with disabilities speak out. *Journal of Aboriginal Health*, *3*(1), 34-43.

*Reason for exclusion:* Not reporting pain-related disability

1. Elias, B., Kaufert, J., Reading, J., O’Neil, J. D., Fricke, M., McDonald, G., & Thibault, J. (1999). Activity limitation and the need for continuing care. *First Nations and Inuit Regional Health Survey. Ottawa, Canada: National Steering Committee*, 137-139.

*Reason for exclusion:* Not reporting pain-related disability

1. Ferucci, E. D. (2008). Arthritis in indigenous populations: a neglected health disparity. *The Journal of Rheumatology*, *35*(6), 956-957.

*Reason for exclusion:* Not an epidemiological study, qualitative or mixed-methods

1. Fey, C. *Listening to Megan: Narratives of a female aboriginal youth with a disability.* (2002).

(Master's thesis report, University of Saskatchewan). <https://saskschoolboards.ca/wp-content/uploads/02-06.htm#Chapter%20One>

*Reason for exclusion:* Not reporting pain-related disability

1. Fricke, M. (1998). Self-determination: the panacea for Canadian aboriginal people with disabilities?. *International Journal of Circumpolar Health*, *57*, 719-724.

*Reason for exclusion:* Not an epidemiological study, qualitative or mixed-methods

1. Goyal, S., Temple, V., Sawanas, C., & Brown, D. (2020). Cognitive profile of adults with intellectual disabilities from indigenous communities in Ontario, Canada. *Journal of Intellectual & Developmental Disability*, *45*(1), 59-65. DOI: 10.3109/13668250.2018.1470160

*Reason for exclusion:* Not reporting pain-related disability

1. Hahmann, T. (2021). *Changes to health, access to health services, and the ability to meet financial obligations among Indigenous people with long-term conditions or disabilities since the start of the COVID-19 pandemic*. Statistics Canada.

*Reason for exclusion:* Not reporting pain-related disability

1. Hamilton, M. K. (1990). The Health and Activity Limitation Survey. Disabled aboriginal persons in Canada. *Health Reports*, *2*(3), 279-287.

*Reason for exclusion:* Not reporting pain-related disability

1. Hamilton, S. (2008). *Work-life balance for parents with low incomes*. (Master’s thesis, University of Northern British Columbia).

*Reason for exclusion:* Not reporting pain-related disability

1. Hop Wo, N. K., Anderson, K. K., Wylie, L., & MacDougall, A. (2020). The prevalence of distress, depression, anxiety, and substance use issues among Indigenous post-secondary students in Canada. *Transcultural Psychiatry*, *57*(2), 263-274. DOI: 10.1177/1363461519861824

*Reason for exclusion:* Not reporting pain-related disability

1. Janzen, T. M., Saklofske, D. H., & Das, J. P. (2013). Cognitive and reading profiles of two samples of Canadian First Nations children: Comparing two models for identifying reading disability. *Canadian Journal of School Psychology*, *28*(4), 323-344. <https://dx.doi.org/10.1177/0829573513507419>

*Reason for exclusion:* Not reporting pain-related disability

1. Knudson, S., Bird-Naytowhow, K., & Baldhead Pearl, T. (2021). Finding our ‘good way’: critical reflections on researching disability, connection, and community from an Indigenous perspective. *Reflective Practice*, *22*(3), 306-318. DOI: 10.1080/14623943.2021.1878124

*Reason for exclusion:* Not an epidemiological study, qualitative or mixed-methods

1. Komenda, P., Lavallee, B., Ferguson, T. W., Tangri, N., Chartrand, C., McLeod, L., ... & Rigatto, C. (2016). The prevalence of CKD in rural Canadian Indigenous peoples: results from the First Nations Community Based Screening to Improve Kidney Health and Prevent Dialysis (FINISHED) screen, triage, and treat program. *American Journal of Kidney Diseases*, *68*(4), 582-590. DOI:
   10.1053/j.ajkd.2016.04.014

*Reason for exclusion:* Not reporting pain-related disability

1. Lamoureux-Tremblay, V., Muckle, G., Maheu, F., Jacobson, S. W., Jacobson, J. L., Ayotte, P., ... & Saint-Amour, D. (2020). Risk factors associated with developing anxiety in Inuit adolescents from Nunavik. *Neurotoxicology and teratology*, *81*, 106903. <https://dx.doi.org/10.1016/j.ntt.2020.106903>

*Reason for exclusion:* Not reporting pain-related disability

1. Larson, C., Pelchat, Y., & Trepanier, L. (1988). Neuromotor disabilities within the James Bay Cree pediatric population. *Arctic Medical Research*, *47*(2), 62-66.

*Reason for exclusion:* Not reporting pain-related disability

1. Latimer, M., Finley, G. A., Rudderham, S., Inglis, S., Francis, J., Young, S., & Hutt-MacLeod, D. (2014). Expression of pain among Mi’kmaq children in one Atlantic Canadian community: a qualitative study. *Canadian Medical Association Open Access Journal*, *2*(3), E133-E138.
   <https://dx.doi.org/10.9778/cmajo.20130086>

*Reason for exclusion:* Not reporting the prevalence, incidence, or factors/characteristics associated with pain-related disability

1. Latimer, M., Simandl, D., Finley, A., Rudderham, S., Harman, K., Young, S., ... & Francis, J. (2014). Understanding the impact of the pain experience on Aboriginal children's wellbeing: Viewing through a two-eyed seeing lens. *First Peoples Child & Family Review*, *9*(1), 22-37.

*Reason for exclusion:* Not an epidemiological study, qualitative or mixed-methods

1. Latimer, M., Rudderham, S., Lethbridge, L., MacLeod, E., Harman, K., Sylliboy, J. R., ... & Finley, G. A. (2018). Occurrence of and referral to specialists for pain-related diagnoses in First Nations and non–First Nations children and youth. *Cmaj*, *190*(49), E1434-E1440.

*Reason for exclusion:* Not reporting pain-related disability

1. Latimer, M., Sylliboy, J. R., MacLeod, E., Rudderham, S., Francis, J., Hutt-MacLeod, D., ... & Finley, G. A. (2018). Creating a safe space for First Nations youth to share their pain. *Pain reports*, *3*(7), e682. DOI: 10.1097/pr9.0000000000000682

*Reason for exclusion:* Not reporting the prevalence, incidence, or factors/characteristics associated with pain-related disability

1. Latimer, M., Sylliboy, J. R., Francis, J., Amey, S., Rudderham, S., Finley, G. A., ... & Paul, K. (2020). Co‐creating better healthcare experiences for First Nations children and youth: The FIRST approach emerges from Two‐Eyed seeing. *Paediatric and Neonatal Pain*, *2*(4), 104-112. <https://dx.doi.org/10.1002/pne2.12024>

*Reason for exclusion:* Not reporting pain-related disability

1. Lawal, M. A., Shalaby, R., Chima, C., Vuong, W., Hrabok, M., Gusnowski, A., ... & Agyapong, V. I. (2021). COVID-19 pandemic: stress, anxiety, and depression levels highest amongst indigenous peoples in Alberta. *Behavioral Sciences*, *11*(9), 115. <https://dx.doi.org/10.3390/bs11090115>

*Reason for exclusion:* Not reporting pain-related disability

1. Lemstra, M., Neudorf, C., Mackenbach, J., D'Arcy, C., Scott, C., Kershaw, T., & Nannapaneni, U. (2008). Risk indicators for depressed mood in youth: Limited association with Aboriginal cultural status. *Paediatrics & child health*, *13*(4), 285-290. <http://dx.doi.org/10.1093/pch/13.4.285>

*Reason for exclusion:* Not reporting pain-related disability

1. Ling, N. M., Loadman, A. E., & Postl, B. D. (1988). Health associated risk factors for academic achievement in a group of Manitoba Indian children. *Arctic medical research*, *47*, 521-525.

*Reason for exclusion:* Not reporting pain-related disability

1. Marrie, R. A., Leung, S., Yu, N., & Elliott, L. (2018). Lower prevalence of multiple sclerosis in First Nations Canadians. *Neurology: Clinical Practice*, *8*(1), 33-39. DOI:10.1212/CPJ.0000000000000418

*Reason for exclusion:* Not reporting pain-related disability

1. Meatherall, B. L., Garrett, M. R., Kaufert, J., Martin, B. D., Fricke, M. W., Arneja, A. S., ... & Embil, J. M. (2005). Disability and quality of life in Canadian aboriginal and non-aboriginal diabetic lower-extremity amputees. *Archives of physical medicine and rehabilitation*, *86*(8), 1594-1602.
   <https://dx.doi.org/10.1016/j.apmr.2004.11.026>

*Reason for exclusion:* Not reporting pain-related disability

1. Mehl-Madrona, L., & Mainguy, B. (2015). Mental disabilities in an aboriginal context. Journal of

Social Work in Disability & Rehabilitation. DOI: 10.1080/1536710X.2015.1068258

*Reason for exclusion:* Not an epidemiological study, qualitative or mixed-methods

1. Anderson, K. (2005). Minobimadziwin: The good life for Aboriginal women. *Centres of Excellence for Women's Health Research Bulletin*, *4*(2), 8.

*Reason for exclusion:* Not an epidemiological study, qualitative or mixed-methods

1. Moore, J. A. (1999). Comparison of risk of conductive hearing loss among three ethnic groups of Arctic audiology patients. *Journal of speech, language, and hearing research*, *42*(6), 1311-1322.
   <http://dx.doi.org/10.1044/jslhr.4206.1311>

*Reason for exclusion:* Not reporting pain-related disability

1. Nagaraj, S., Barnabe, C., Schieir, O., Pope, J., Bartlett, S. J., Boire, G., ... & Canadian Early Arthritis Cohort Study Investigators. (2018). Early rheumatoid arthritis presentation, treatment, and outcomes in aboriginal patients in Canada: a Canadian early arthritis cohort study analysis. *Arthritis Care & Research*, *70*(8), 1245-1250. <https://dx.doi.org/10.1002/acr.23470>

*Reason for exclusion:* Not reporting pain-related disability

1. Nasreen, S., Brar, R., Brar, S., Maltby, A., & Wilk, P. (2018). Are Indigenous determinants of health associated with self-reported health professional-diagnosed anxiety disorders among Canadian First Nations adults?: Findings from the 2012 Aboriginal Peoples Survey. *Community mental health journal*, *54*, 460-468. DOI: 10.1007/s10597-017-0165-0

*Reason for exclusion:* Not reporting pain-related disability

1. Nelson, C., Lawford, K. M., Otterman, V., & Darling, E. K. (2018). Mental health indicators among pregnant Aboriginal women in Canada: results from the Maternity Experiences Survey. *Health Promotion and Chronic Disease Prevention in Canada*, *38*(7).
   <https://dx.doi.org/10.24095/hpcdp.38.7/8.01>

*Reason for exclusion:* Not reporting pain-related disability

1. Newbold, K.B. (1999). Disability and use of support services within the Canadian aboriginal population. *Health and Social Care in the Community, 7*(4), 291–300. DOI: 10.1046/j.1365-2524.1999.00189.x

*Reason for exclusion:* Not reporting pain-related disability

1. Newbold, K. B., & Simone, D. (2015). Comparing disability amongst immigrants and native-born in Canada. *Social Science & Medicine*, *145*, 53-62. <https://dx.doi.org/10.1016/j.socscimed.2015.09.035>

*Reason for exclusion:* Non-Indigenous populations and not in Canada

1. Ng, E. (1996). Disability among Canada's aboriginal peoples in 1991. *Health Reports*, *8*(1), 25-32. *Reason for exclusion:* Not reporting pain-related disability
2. Ng, C., Kue Young, T., & Chatwood, S. (2010). Arthritis in the Canadian Aboriginal Population: North-South Differences in Prevalence and Correlates. *Chronic Diseases in Canada, 31*(1), 22-26.

*Reason for exclusion:* Not reporting pain-related disability

1. Perreault, K., Dufresne, P., Potvin, L., & Riva, M. (2023). Housing as a determinant of Inuit mental health: Associations between improved housing measures and decline in psychological distress after rehousing in Nunavut and Nunavik. *Canadian Journal of Public Health*, *114*(2), 241-253. DOI: 10.17269/s41997-022-00701-0

*Reason for exclusion:* Not reporting pain-related disability

1. Robison, J. (2003). *Disability... it's not in me... it's out there. A comparative ethnography of environmental factors influencing participation in three Baffin Island communities*. (Master’s thesis, Queen’s University).

*Reason for exclusion:* Not reporting pain-related disability

1. Saeedi, J., Rieckmann, P., Yee, I., Tremlett, H., & UBC MS clinic neurologists. (2012). Characteristics of multiple sclerosis in aboriginals living in British Columbia, Canada. *Multiple Sclerosis Journal*, *18*(9), 1239-1243. <https://dx.doi.org/10.1177/1352458512436595>

*Reason for exclusion:* Not reporting pain-related disability

1. Shackel, D. W. (2008). The experience of First Nations people with disabilities and their families in receiving services and supports in First Nations communities in Manitoba: honouring the stories. (Master’s thesis, University of Manitoba).

*Reason for exclusion:* Not reporting pain-related disability

1. Stamos-Destounis, B. (1993). Pediatric impairments in Canada's Arctic. (Master’s thesis, McGill University).

*Reason for exclusion:* Not reporting pain-related disability

1. Svenson, L. W., Warren, S., Warren, K. G., Metz, L. M., Patten, S. B., & Schopflocher, D. P. (2007). Prevalence of multiple sclerosis in First Nations people of Alberta. *Canadian journal of neurological sciences*, *34*(2), 175-180. <https://doi.org/10.1017/S0317167100006004>

*Reason for exclusion:* Not reporting pain-related disability

1. Tjepkema, M. (2002). The health of the off-reserve Aboriginal population [Canadian Community Health Survey-2002 Annual report]. *Health reports*, *13*, 73.

*Reason for exclusion:* Not reporting pain-related disability

1. Tripp, D. A., VanDenKerkhof, E. G., & McAlister, M. (2006). Prevalence and determinants of pain and pain‐related disability in urban and rural settings in southeastern Ontario. *Pain Research and Management*, *11*(4), 225-233.

*Reason for exclusion:* Non-Indigenous populations and not in Canada

1. Tutty, L. M., Radtke, H. L., Thurston, W. E., Nixon, K. L., Ursel, E. J., Ateah, C. A., & Hampton, M. (2020). The mental health and well-being of Canadian Indigenous and non-Indigenous women abused by intimate partners. *Violence Against Women*, *26*(12-13), 1574-1597. DOI: 10.1177/1077801219884123

*Reason for exclusion:* Not reporting pain-related disability

1. VanEvery, R., Latimer, M., & Naveau, A. (2022). Clinical strategies to develop connections, promote health and address pain from the perspectives of Indigenous youth, Elders, and clinicians. *Frontiers in Pain Research*, *3*, 857624. <https://dx.doi.org/10.3389/fpain.2022.857624>

*Reason for exclusion:* Not reporting pain-related disability

1. Vang, Z. M., Chau, S., Kobayashi, K. M., Owen, M. J., McKenzie-Sampson, S., Mayrand-Thibert, J., & Brass, G. M. (2024). Pain and functional limitations among midlife and older Canadians: The role of discrimination, race, and sense of belonging. *The Journals of Gerontology, Series B: Psychological Sciences and Social Sciences*, *79*(4). <https://dx.doi.org/10.1093/geronb/gbab137>

*Reason for exclusion:* Not reporting pain-related disability

1. Varcoe, C., Ford-Gilboe, M., Browne, A. J., Perrin, N., Bungay, V., McKenzie, H., ... & Dion Stout, M. (2021). The efficacy of a health promotion intervention for Indigenous women: Reclaiming our spirits. *Journal of interpersonal violence*, *36*(13-14). DOI: 10.1177/0886260518820818

*Reason for exclusion:* Not reporting the prevalence, incidence, or factors/characteristics associated with pain-related disability

1. Wee, J. (2009). Creating a registry of needs for persons with disabilities in a Northern Canadian community: the disability registry project. *Asia Pacific Disability Rehabilitation Journal*, *20*, 1-18.

*Reason for exclusion:* Not reporting the prevalence, incidence, or factors/characteristics associated with pain-related disability

1. Wilson, K., & Cardwell, N. (2012). Urban Aboriginal health: Examining inequalities between Aboriginal and non-Aboriginal populations in Canada. *The Canadian Geographer*, *56* (1), 98-116. DOI: 10.1111/j.1541-0064.2011.00397.x

*Reason for exclusion:* Not reporting pain-related disability

**Appendix D: Preferred Reporting Items for Systematic reviews and Meta-Analyses extension for Scoping Reviews (PRISMA-ScR) Checklist**

| **SECTION** | **ITEM** | **PRISMA-ScR CHECKLIST ITEM** | **REPORTED ON PAGE #** |
| --- | --- | --- | --- |
| **TITLE** | | | |
| Title | 1 | Identify the report as a scoping review. | 1 |
| **ABSTRACT** | | | |
| Structured summary | 2 | Provide a structured summary that includes (as applicable): background, objectives, eligibility criteria, sources of evidence, charting methods, results, and conclusions that relate to the review questions and objectives. | 1 |
| **INTRODUCTION** | | | |
| Rationale | 3 | Describe the rationale for the review in the context of what is already known. Explain why the review questions/objectives lend themselves to a scoping review approach. | 2 |
| Objectives | 4 | Provide an explicit statement of the questions and objectives being addressed with reference to their key elements (e.g., population or participants, concepts, and context) or other relevant key elements used to conceptualize the review questions and/or objectives. | 4-5 |
| **METHODS** | | | |
| Protocol and registration | 5 | Indicate whether a review protocol exists; state if and where it can be accessed (e.g., a Web address); and if available, provide registration information, including the registration number. | 6 |
| Eligibility criteria | 6 | Specify characteristics of the sources of evidence used as eligibility criteria (e.g., years considered, language, and publication status), and provide a rationale. | 5-6 |
| Information sources* | 7 | Describe all information sources in the search (e.g., databases with dates of coverage and contact with authors to identify additional sources), as well as the date the most recent search was executed. | 6-7 |
| Search | 8 | Present the full electronic search strategy for at least 1 database, including any limits used, such that it could be repeated. | Appendix A |
| Selection of sources of evidence† | 9 | State the process for selecting sources of evidence (i.e., screening and eligibility) included in the scoping review. | 7 |
| Data charting process‡ | 10 | Describe the methods of charting data from the included sources of evidence (e.g., calibrated forms or forms that have been tested by the team before their use, and whether data charting was done independently or in duplicate) and any processes for obtaining and confirming data from investigators. | 7-8 |
| Data items | 11 | List and define all variables for which data were sought and any assumptions and simplifications made. | 7-8 |
| Critical appraisal of individual sources of evidence§ | 12 | If done, provide a rationale for conducting a critical appraisal of included sources of evidence; describe the methods used and how this information was used in any data synthesis (if appropriate). | N/A |
| Synthesis of results | 13 | Describe the methods of handling and summarizing the data that were charted. | 8 |
| **RESULTS** | | | |
| Selection of sources of evidence | 14 | Give numbers of sources of evidence screened, assessed for eligibility, and included in the review, with reasons for exclusions at each stage, ideally using a flow diagram. | 10 and Figure 1 |
| Characteristics of sources of evidence | 15 | For each source of evidence, present characteristics for which data were charted and provide the citations. | Table 1 |
| Critical appraisal within sources of evidence | 16 | If done, present data on critical appraisal of included sources of evidence (see item 12). | N/A |
| Results of individual sources of evidence | 17 | For each included source of evidence, present the relevant data that were charted that relate to the review questions and objectives. | 10-12 |
| Synthesis of results | 18 | Summarize and/or present the charting results as they relate to the review questions and objectives. | 10-12 |
| **DISCUSSION** | | | |
| Summary of evidence | 19 | Summarize the main results (including an overview of concepts, themes, and types of evidence available), link to the review questions and objectives, and consider the relevance to key groups. | 12 |
| Limitations | 20 | Discuss the limitations of the scoping review process. | 13-14 |
| Conclusions | 21 | Provide a general interpretation of the results with respect to the review questions and objectives, as well as potential implications and/or next steps. | 14-15 |
| **FUNDING** | | | |
| Funding | 22 | Describe sources of funding for the included sources of evidence, as well as sources of funding for the scoping review. Describe the role of the funders of the scoping review. | Title page |

JBI = Joanna Briggs Institute; PRISMA-ScR = Preferred Reporting Items for Systematic reviews and Meta-Analyses extension for Scoping Reviews.

* Where *sources of evidence* (see second footnote) are compiled from, such as bibliographic databases, social media platforms, and Web sites.

† A more inclusive/heterogeneous term used to account for the different types of evidence or data sources (e.g., quantitative and/or qualitative research, expert opinion, and policy documents) that may be eligible in a scoping review as opposed to only studies. This is not to be confused with *information sources* (see first footnote).

‡ The frameworks by Arksey and O’Malley (6) and Levac and colleagues (7) and the JBI guidance (4, 5) refer to the process of data extraction in a scoping review as data charting*.*

§ The process of systematically examining research evidence to assess its validity, results, and relevance before using it to inform a decision. This term is used for items 12 and 16 instead of "risk of bias" (which is more applicable to systematic reviews of interventions) to include and acknowledge the various sources of evidence that may be used in a scoping review (e.g., quantitative and/or qualitative research, expert opinion, and policy document).

*From:* Tricco AC, Lillie E, Zarin W, O'Brien KK, Colquhoun H, Levac D, et al. PRISMA Extension for Scoping Reviews (PRISMAScR): Checklist and Explanation. Ann Intern Med. 2018;169:467–473. [doi: 10.7326/M18-0850](http://annals.org/aim/fullarticle/2700389/prisma-extension-scoping-reviews-prisma-scr-checklist-explanation).
